# RUDO: A Home Ambient Intelligence System for Blind People

**DOI:** 10.3390/s17081926

**Published:** 2017-08-22

**Authors:** Milan Hudec, Zdenek Smutny

**Affiliations:** 1 Department of Computer Science, Faculty of Natural Sciences, Matej Bel University, Tajovského 40, 974 01 Banská Bystrica, Slovakia; milan.hudec@umb.sk; 2 Department of Systems Analysis, Faculty of Informatics and Statistics, University of Economics, Prague, W. Churchill Sq. 1938/4, 130 67 Prague, Czech Republic

**Keywords:** ambient assisted living, artificial neural network, blind people, Key RollOver, speech synthesis, user interface, security, work with computer, Z-Wave, zone regulation

## Abstract

The article introduces an ambient intelligence system for blind people which besides providing assistance in home environment also helps with various situations and roles in which blind people may find themselves involved. RUDO, the designed system, comprises several modules that mainly support or ensure recognition of approaching people, alerting to other household members’ movement in the flat, work on a computer, supervision of (sighted) children, cooperation of a sighted and a blind person (e.g., when studying), control of heating and zonal regulation by a blind person. It has a unified user interface that gives the blind person access to individual functions. The interface for blind people offers assistance with work on a computer, including writing in Braille on a regular keyboard and specialized work in informatics and electronics (e.g., programming). RUDO can complement the standard aids used by blind people at home, it increases their independence and creates conditions that allow them to become fully involved. RUDO also supports blind people sharing a home with sighted people, which contributes to their feeling of security and greater inclusion in society. RUDO has been implemented in a household for two years, which allows an evaluation of its use in practice.

## 1. Introduction

The development of ambient intelligence (AmI) technologies creates new ways of caring for people with disadvantages, which includes supporting them in everyday activities and social integration. The group of disabled people includes the blind, for whom designed the RUDO system is intended. According to a World Health Organization’s report [1], the total number of blind people in the world is estimated at 39 million. Of this number 82% are people over 50 years of age, and 41% of all blind people live in either India or China. Despite the fact that this amounts to only about 0.6% of the world population, there has been active research in this area, mainly design type of research (i.e., the design of aids for blind people).

The aim of this article is therefore to present the design of such an AmI home system for blind people, based on the perceived needs of a blind person. The described system was realized as a functional prototype and implemented in a blind person’s household, which makes it possible to discuss the experience so far, the advantages as well as the limitations of the system, and the possibilities of its further extension. This system is called RUDO and it is designed for a blind person’s stay in a home environment, during which it supports their basic everyday needs, and suitably complements the standard tools used by a blind person in a household. The AmI system RUDO is designed to support people who are completely blind and it does not take into consideration the specific needs of people with low vision, who number over six times more than blind people [1]. It should be added that the principal designer of RUDO system and first author of the article is blind himself, which made it possible to sufficiently test this system in practice and to eliminate the flaws that were discovered along the way.

The development of RUDO system was gradual, with work on the first module starting as early as the year 2000—see the history of development in Section 5. This article presents its fourth version as a functional unit comprising individual modules. The current version of RUDO system offers home assistance mainly in the following areas: recognition of incoming people and notification about movement in the flat, working on a computer, including specialized work in electrotechnics and informatics, supporting the cooperation of a blind/sighted child with a blind/sighted parent during preparation for school on a computer, basic support of child supervision by a blind parent, two-phase heating regulation operated by a blind user. For this purpose, RUDO uses a unified user interface that allows a blind user to operate the individual parts, and special applications and auxiliary scripts.

The article builds on design science methodology and an iterative approach to designing solutions—the so-called engineering cycle, see [2]—in connection with the defined problem context of the presence of a blind person in a home environment. According to design science methodology [2], a research question should be posed that concerns the design of a new artefact—in the case of this article it is the AmI system RUDO. The research question (or also technical research problem) is: how to design an AmI system that would satisfy the needs of blind people in home environment so that they would be able to perform everyday domestic activities more independently, and would also contribute to blind people’s social inclusion in home environment?

This article is structured as follows: Section 2 presents related work and the state of research. Section 3 presents the methodology used in the development of the system. Section 4 introduces the problems and needs of a blind person during their stay in home environment which the designed system has to fulfil. Section 5 introduces the design of RUDO. Section 6 contains a discussion of experiences using the RUDO prototype in practice. A conclusion with the possibilities of further development of AmI systems for blind people is to be found in Section 7.

## 2. Related Work

It seems appropriate to begin with an overview of the current state of research and to define on that basis the research area into which the design of RUDO system belongs. Applied research and development focused on assistive technology can be divided into two complementary directions: aid design and AmI environments or smart environments (SmE) creation. These two directions are discussed in the first two subsections. The third subsection deals with the individual topics related to the functional components of RUDO system.

### 2.1. Design of Aids

In the field of designing aids that would facilitate to a blind person new possibilities of interaction in physical environment, a great deal of attention has been given to compensating for the lack of sight in everyday activities (e.g., recognizing objects, people, movement in space). Current research and development focuses mainly on two sub-fields. The first sub-field is designing aids for helping blind people to find [3] and recognize indoor objects [4,5]. Connected to that is also the design of aids that support user’s independence while their use (wearing) does not cause the user any discomfort [6]. The second sub-field is creating aids for detecting obstacles or barriers and for helping blind people navigate both inside and outside buildings using various locating and other supporting technologies. Articles describe, e.g., the use of GPS [7,8], WiMAX [9], 3-D sound, or a magnetic compass and gyroscope [10]. Static object detection from images is used for avoiding obstacles [11,12], along with ultrasonic sensors [13,14,15,16], or other sensors using a GIS platform for navigation [17,18]. The latest assistive technologies in development try to offer both functions, i.e., object recognition and navigation, mainly in indoor environments, see e.g., [5].

A specific sub-field is creating aids for operating information and communication technologies such as computers or mobile phones [19,20], which in turn make it possible for blind people to be in Internet-mediated environments and which improve social inclusion. This is the direction taken for example in the development of universal audio and tactile user interfaces for using computing technology [21,22].

Relevant behavioural studies complementing the design type of research focus mainly on the capacities that blind people have for compensating for the loss of sight, and on their spatial orientation. Such studies focus for example on the possibility of mutual navigation of a blind person by another blind person via phone [23], spatial representations that blind people use when they move in some space [24], the use of echolocation by the blind and the advantages it brings them in real life [25,26], or the everyday challenges that blind people have to face [27].

### 2.2. Creation of SmE and AmI Environments

The other direction is connected with the possibility of creating SmE or AmI environments in which a person could stay, and this environment would fulfil the defined vital needs of this person [28]. Among such systems is the sub-field of ambient assisted living (AAL) that focuses on supporting the provision of user-dependent services for elderly and disabled people [29,30]. This direction is linked to the development of intelligent and easily connectible information and communication technologies, which can be suitably assembled and used for the purpose of improving the quality of a disadvantaged person’s life. AAL systems installed in the interior of buildings for the purpose of assisting residents—for an overview see e.g., [31,32,33,34]—are in the vast majority of cases focused on helping the elderly [35,36]. This means that they focus mainly on assisting people with musculoskeletal disorders [37,38], low vision [39], less severe forms of senile dementia [40], and on maintaining hygiene and improving safety [41]. It is equally important for the elderly to maintain their social network and to communicate within this network using new technologies [42,43]. These studies have been carried out mainly in connection with the increasing average life expectancy in developed countries—see also the European applied research programme: Active and assisted living [44].

Despite the above, there is only a small number of articles that mention AAL systems not just in connection with elderly people, but also emphasize the need of focusing on physically disabled people [37,38]—including the blind. This unsatisfactory situation is also confirmed by [45]: “Although various technological approaches have been proposed, none of them comprehensively addresses assistance for daily activities and social inclusion of totally blind and visually impaired people.” This article further points out that the current trend is designing solutions focused on one specific kind of support (e.g., aid for navigation). Unfortunately, these technical solutions are often not based on the specific requirements of blind users and therefore are not accepted by these users in practice [45]. This discrepancy between the design of technical solutions and their implementation in a social context is a future challenge that needs to be addressed. In this respect, the design and the realization of RUDO system is unique, as it was carried out directly by a blind person.

The topic of deploying AAL systems and aids in practice is also relevant to social informatics, which deals with the issue of understanding the relations between the design and the use of ICT in a particular social context. This field complements the design type of research and enables a continuous improvement of the designed solutions to discovered problems. Especially in German-speaking countries, social informatics is focused on the study and the improvement of the quality of life of both individuals and specific social groups (e.g., disabled people) [46,47].

Despite the fact that this article focuses on a specific group of disadvantaged people, it cannot be ignored that 82% (32 million) of blind people are over 50 years of age, which may lead to limitations in terms of user interface. As regards elderly people, there might be difficulties related to their refusal or inability to use new technologies, which in this social group may be aggravated by their handicap. Elderly people prefer familiar patterns, very simple control [48], and their attitude towards new technologies is influenced by their experience with computers and their conviction about the usefulness of learning to use new technologies [49]. For these reasons, too, it is more logical to design for blind people SmE and AmI environments and aids using a unified interface that enables a number of activities, rather than developing a large number of single-purpose tools, each with their own interface—see also [5,17]. SmE and AmI environments allow a greater technological independence of the individual parts (modules), which makes it possible to modify and improve individual technological elements of a system integrated in an interface without the need for (major) changes to the user interface, which would take a blind person too long to get used to.

Based on an extensive research of available literature in Web of Science and Scopus databases, it can be said that there is not much that has been done in the field of AmI or AAL system design for blind people—this conclusion is also confirmed by [45]. It is possible to find articles that present the possibility of creating an AmI environment for blind people [22], but no detailed designs have yet been introduced, nor any tested prototypes of complex systems.

This article fills the gap in the area of assisting the blind in home environment by designing a home AmI system for blind people which is based on their needs and which comprehensively addresses assistance for daily activities and social inclusion of the blind. This system needs to be understood as a whole; but since no other system offers all these services, the following subsection introduces similar solutions in the individual areas in which RUDO offers home assistance. At the same time, RUDO’s unique functions are highlighted.

### 2.3. Related Work in Areas Provided by RUDO System

This subsection focuses on four aspects of RUDO system and accentuates those functions that are unique in comparison with other existing solutions. The four aspects are: recognizing incoming persons and providing information about movement in the flat; working on computer including specialized work in electrotechnics and informatics; basic support for child supervision by a blind parent; two-phase heating regulation operated by a blind user.

#### 2.3.1. Recognizing Incoming Persons and Notification about Movement in the Flat

As mentioned in Section 2.1, research focusing on aids for the blind concentrates mainly on object recognition and the navigation of a blind person in a certain environment. Papers [50,51,52] discuss the design of tools for person recognition with a focus on visually impaired people. They use face recognition technology for this purpose. This person recognition technology can easily be transferred to the context of blind people’s home environment, on which this article focuses. Unlike the above, RUDO uses information about people’s movement for their recognition, rather than their faces. A unique function of RUDO system is the information it provides about the movement of people in the flat, which—together with person recognition—creates for the blind person a whole new communication context in home environment.

#### 2.3.2. Working on Computer Including Specialized Work in Electrotechnics and Informatics

Researchers started to explore the possibility of involving computers in the jobs of the blind as early as the 1960s [53], but it was only with the development of personal computers, their ready availability and the transition from the command line to a graphic user interface that new challenges connected with operating computers and other modern technologies started to emerge. The current research and development of assistive technologies, adaptive systems and context-aware applications focuses on such technologies that make daily living easier for people with special needs, including the blind [54]. This includes work with computer, for which various solutions have been designed that use text-to-speech or haptic interfaces for blind computer users [54,55]. In practice, this means using a speech synthesizer and a keyboard or another special haptic device. In this decade, emphasis has been put on adaptive technologies, which can adjust to different types of users (motor-impaired, blind, deaf), but their disadvantage is that they are often based on a visually oriented interface. In order to create user interfaces for computer applications that would be accessible to the blind, it is vital to understand their mental model of the world [56]. This representation of the world in which they live and interact can be transformed into general principles and reflected in computer-generated virtual environments. That is why in the case of the blind there are specific approaches to the design of user interfaces based on specific usability requirements that are unnecessary for sighted users [57,58].

The user interface offered by RUDO is not an adaptive technology; on the contrary, it focuses only on blind users, to whom it offers two user interfaces in the form of a command line and a textual semi-graphic mode. The semi-graphic environment for blind people is offered by a number of applications for OS Linux. The most commonly used applications for normal work on computer include e.g., the e-mail client Alpine or web browsers Links and W3M. RUDO’s user interface is based on the known principles of operating computer applications to which the blind are accustomed and which correspond with their mental model of the world. A standard keyboard in the so-called Braille mode is used for input. A special Braille driver for ordinary keyboards enables writing even the special Slovak characters (8-dot Braille) and switching between normal mode for the sighted and Braille mode for the blind. This input can be replaced by a more expensive Braille keyboard. The output uses a speech synthesizer and a refreshable Braille display. These are standard tools used for the interaction between a blind person and a computer.

As early as the 1960s, it was supposed that intelligent blind people could be involved as programmers, as they had a trained memory and object imagination [53]. Current research, too, focuses on improving the teaching of programming and other technological skills of blind people [59,60,61]. A part of RUDO system is the ROWS module that enables normal work with computer, e.g., creating formatted documents, programming, and it offers other supporting scripts and assistance software to speed up work. The ROWS module is unique because it enables a blind person to do advanced work in the field of informatics and electronics focusing on natural language processing, genetic algorithms and neural networks, and to measure electrical quantities, ranges and values by a multimeter. ROWS module can also be installed on a computer on its own and thus it offers full mobility between working at home and in a workplace.

#### 2.3.3. Basic Support of Child Supervision by a Blind Parent

Not a single source was found that considered the issue of supporting child supervision by a blind parent from the perspective of a blind parent, neither in a behavioural nor in a design type of research. Studies dealing with child supervision tend to focus rather on the perspective of the child. For instance, the behaviour and subsequent injury of a child in relation to the quality of supervision [62], or the research of the behaviour of autistic children and how the intensity of supervision affects their development [63]. In the area of blind children research, there are mainly articles dealing with their tuition, e.g., spatial orientation with the use of vibration [64]. RUDO offers assistance during child supervision by a blind parent, but this is not a function that would be emphasized when designing the overall solution. On the contrary, it is a solution given by a need in a specific social context in which a prototype of the AmI system was to be installed. The suggested solution has serious limitations and offers only elementary support for child supervision inside and outside the house (in the garden) in the form of informational sounds that provide notification about the movement of the child in the flat, and acoustic feedback from the garden.

#### 2.3.4. Heating Regulation Operated by a Blind User

Modern thermostats, as well as more complex solutions offering home automatization, also tend to offer a user interface for blind people, see e.g., [65]. From this perspective, that is not a unique function of RUDO. However, it is a unique function in Slovak context (fully supported Slovak language) with extensive options for a blind user to set the two-phase heating process and zonal temperatures. The module that ensures two-phase heating regulation also uses other parts of the RUDO system, e.g., for notifications about heating and temperature via the indoor speaker.

## 3. Methodology

The selected research method is based on design science research (DSR), which focuses on solving a problem by means of the design, construction and evaluation of an artefact. DSR is oriented on solving an identified problem with a view to finding a satisfactory solution that, however, does not necessarily have to be entirely optimal ([66], p. 68). Such research assumes that there is not a single solution, but multiple possible solutions of the problem context ([2], p. 6). Each solution, which is validated by experts or evaluated in practice, contributes to design theory in the given area ([2], pp. 95–102), because the way in which the design of an artefact is carried out (problem-solving theory) is to some extent universal and independent. Contrary to that, the designed artefact is connected with certain knowledge and technologies determined by the time in which the artefact was created (implementing a prototype in practice).

DSR offers a high degree of relevance of the solution (e.g., organizational or social need) with a rigorous approach to problem-solving, using existing knowledge bases and methodological procedures (scientific approach). The reason for choosing design science methodology is the use of design and engineer cycles, which are suitable for an iterative long-term development of a given solution. Each version of RUDO system was evaluated in practice, and it is therefore an independent engineer cycle of solution development. There is a large number of methodological approaches used in DSR and based on design science [2,67]. The vast majority of them use four basic stages of research upon which this article is also founded: (1) identifying and defining a problem, (2) suggestions for possible solutions, (3) development and (4) evaluation ([66], p. 92).

The first stage that focuses on identifying and defining the problem is based on a problem perceived by the researcher, which should then be further supported by exploratory research using primary and secondary sources ([68], p. 72). The researcher should justify the relevance of the research, the importance of the problem, and the applicability of a potential solution [69]. On the basis of research of scientific literature in Section 2, the authors believe that designing and developing a home AmI system for blind people is justified and it is at the same time a rational outcome of technological progress.

The principal designer of RUDO system has been blind since birth. That is why the basic requirements for that system are based on his own needs while later these have been revised and complemented by the needs mentioned by other blind people. Thanks to this direct involvement of the blind person in the design and development of the artefact, it is examined from both an academic and a practical standpoint, which is also typical for DSR ([66], p. 67; [70]) and as a result it can improve the theoretical foundations of the researched area ([71], pp. 33–34).

The authors also built upon their own exploratory research, which included studying available scientific literature and interviewing five blind people—members of the group Slovak Blind and Partially Sighted Union. The interviews were conducted at the beginning of the development of the fourth version of RUDO system to make sure that no important need felt by other blind people in their home environments was omitted. The basic information about the blind respondents is as follows: woman, 60–70 years old, university education, lives alone; man 40–50 years old, university education, lives with sighted wife and child; man, 30–40 years old, university education, lives with sighted wife and child; man, 30–40 years old, university education, lives with sighted wife; man, 30–40 years old, university education, lives alone. The interviews focused on the following areas:

- What compensatory aids do they use at home?
- What compensatory aids would they welcome while being at home?
- For what purposes do they use the assistance of the sighted person at home?
- If they are parents, what needs do they have when supervising the children at home?

The results are formulated in Section 4.1, which also introduces other problems that the blind have to face in connection with the implementation of new technologies. The identified problems are used as a basis for defining what blind people require of the designed home AmI system.

The next two stages, focusing on the design and development of RUDO system, are presented in Section 5. The aim of this part is to describe the designed system with such detail that would allow any researcher (or company) to replicate it or implement its modules in their own environment or solution (because each implementation is unique). This presupposes that different but functionally similar technologies can be used as long as the main features of the system are preserved. In other words, this means that the implemented system should fulfil the defined range of needs in the same way as RUDO system and thus solve the problems perceived by blind people.

The prototype of the described system is practically demonstrated and evaluated by its implementation in the home of a blind person. The version of RUDO system introduced here has been implemented in the home for a period of two years; the experience from that implementation is described in Section 6, which also includes further relevant discussion. Section 3.1 introduces in detail the method used for the evaluation of the designed artefact.

### 3.1. Evaluation Methodology

While ex-ante evaluation (or also validation) is limited only to the evaluation of a designed artefact by selected specialists in the field and by the interested parties without necessarily implementing or using the artefact ([72], p. 138), ex post evaluation judges the artefact that is already placed into a relevant problem context (e.g., a particular institution). The latter evaluation method involves considerable costs related to the realization of such research—see e.g., [73]. This gave rise to new methods of artefact evaluation, mainly in the field researching information systems and technologies, which facilitate the transition from an academic design into practice with its respective problem context in which the artefact is applied. The research methods used in such artefact evaluation often connect the principles of action research (AR) and DSR [74]. An example of this method is action design research (ADR), which is closer to practical ex-post evaluation [75], or technical action research (TAR), which is closer to ex ante evaluation [76].

The approach chosen for the evaluation of RUDO system is based on ADR. For this purpose, a fully functional prototype of RUDO was installed in the blind person’s home, and the researcher who had designed the system was also involved in the evaluation of the artefact. This approach is more typical for ex ante evaluation—for example in TAR method evaluation [76] a researcher can take on several roles: technical researcher, empirical researcher, and helper.

ADR evaluation focuses on the stages preceding the production version of a system, and at the same time it is part of the engineering cycle in design science, which enables further iterative improvement of the designed artefact ([2], p. 31). ADR evaluation is based on the principles applied in AR, which is a qualitative type of research that is generally accepted also within DSR ([66], p. 22; [72], p. 39; [2], p. 217). The same basis is used in the evaluation of RUDO system, which is based on the subjective observations of the users of the installed RUDO prototype (sighted and blind family members). Another reason for choosing a qualitative type of research is the fact that some functions use stochastic and learning approaches used in artificial intelligence (neural network, genetic algorithm). It is therefore difficult to unambiguously determine the system’s success rate in the individual tasks because the system learns continuously (e.g., when identifying incoming people).

The evaluation procedure ([72], p. 137) using feedback from users is as follows: (1) first it focuses on assessing the fulfilment of the individual requests made by blind people on the home AmI system, and then (2) follows an evaluation of selected aspects from the perspectives of sighted and blind users (family members). This was based on the blind person’s notes and an interview with a sighted household member. Finally, the limitations of the designed system and the possibilities of its improvement are discussed.

### 3.2. The Limitations of the Research

The conducted design type of research has several limitations given by two factors. The first factor is the length of development (17 years), which is reflected in two aspects: the increasing complexity of the designed system, and the corresponding technological solutions of some parts of the system. The entire RUDO system, including all supporting programs, has approximately 150,000 lines of code, and the software as well as the hardware was developed by a single person. This fact limits the flexibility of such a system and possible changes. That is why some technological solutions could be, from today’s perspective, approached differently in terms of both hardware and software. The authors point out such shortcomings in the relevant places in the article. On the other hand, it is necessary to consider RUDO system as a whole and to focus on how it fulfils the defined needs of blind people in home environment, because the whole is not merely a sum of its parts. It should also be added that the designed system is intended for people with very low income and that needs to be taken into account when selecting which technologies would be used. This limitation should also be seen from the perspective of design science methodology, which, for this very reason, accentuates not the final artefact that solves the problem context, but rather the way of solving the problem that is not necessarily associated with the knowledge and the technologies of a given period. Technologies can be replaced by new ones, but the basic contextual principles of their use tend to remain.

The other factor is the fact that the prototype was implemented in a single user context, and the subsequent evaluation of the whole system. Due to this restriction, the authors introduced in Section 6 multiple perspectives, from which they evaluated the designed and installed system RUDO. The concluding discussion further specifies how a particular system is customized for various contexts in which blind people may find themselves when at home, which go beyond the single user evaluation that was carried out. Related to the exploratory research and evaluation is also the issue of a broader context, since all the blind respondents were from Slovakia and the prototype was also implemented only in Slovakia, and therefore it was mainly the needs of Slovakian blind people that were considered. Different countries presumably have not only different social inclusion of the blind, but also different state support or availability of care services and aids.

## 4. The Identified Problems and Needs of Blind People in Home Environment

This section follows three goals related to the first stage of the described methodology and the research question. The first goal is to introduce other views on the needs and problems of blind people in their households in Section 4.1. Interviews with five blind people are evaluated here for this purpose. The results also function as an assurance that the principal designer did not omit any important need or problem that blind people experience at home. Following from this part is a consideration of the problems with using the user interface of new household appliances from the perspective of a blind person in Section 4.2. Also introduced here is the topic of the usability of standard keyboards for writing in Braille when using a computer, i.e., without having to use a special Braille keyboard. The above is used in Section 4.3, which defines the areas of assistance that RUDO should provide, with regard to the specific context in which the installation of the prototype for a testing period was planned.

### 4.1. Evaluation of Exploratory Research

When designing any sociotechnical system it is vital to ask questions about the needs of its users [69,77]. Exploratory qualitative preliminary research was therefore carried out for that purpose and it complements the research of available literature. This type of research contributes mainly to a broader understanding and to defining the focus area of the subsequent design type of research. This means mainly understanding the environment surrounding the problem that is being solved in this article by designing an artefact. Exploratory research consisted of short interviews with five blind people—their basic profiles and the questions are presented in Section 3 as part of the methodology.

The aim of these interviews was to enable the principal designer of the system to discuss the perceived problems with other members of his community. They also offer an assurance that the needs and problems identified earlier also concern a wider community of blind people. The outcomes of the interviews were taken into consideration in the latest version 4.0 of the designed system RUDO—see the history of development in Section 5. The interviews led to the following findings about blind people in home environment:

- *What compensatory aids do they use at home?* While staying at home, the respondents use the following tools most often (agreement of 3 or more respondents): computer, printer, music player and clock; and less often (agreement of 2 or fewer respondents) thermometer, blood pressure monitor, level indicator or mobile phone.
- *What compensatory aids would they welcome while being at home?* In terms of compensatory aids, they would most welcome a security system that would detect that someone is coming and would then identify that person. In connection with housework, they would also appreciate a washing machine and a stove with voice feedback and automatic regulation.
- *For what purposes do they use the assistance of the sighted person at home?* At home, they use the assistance of a sighted person most often when they need to read something or find out the current conditions (e.g., temperature, pressure) inside or outside the house.
- *If they are parents*, *what needs do they have when supervising the children at home?* When it is necessary to supervise children, the respondents stated that they need to ascertain on request the occupation and location of the child.

The subsequent discussion with the respondents established specific needs according to other household members—different social context at home. In the respondents’ opinion, a blind person might feel different needs depending on whether they live alone or with someone else. If a blind person lives together with other people, their mutual relationship (spouse, parent, child) is also significant, as is the blind person’s age in terms of ontogenetic development (e.g., the learning needs of blind children and sighted adults are different than those of blind adults and sighted children). For this purpose, introduced below are different social contexts of a household, identified on the basis of further discussion with the respondents and that could be fulfilled by a home AmI system or by existing aids for the blind.

#### 4.1.1. A Blind Person Who Lives Alone

The need for assistance in this case is strongest because the person needs, for example, assistance for reading (from a screen, a newspaper or a computer), orientation in the colours of clothes, operating all household equipment including the heating system, and recognizing objects. In this respect it is vital to receive e.g., auditory feedback from the things that are being used or from the home AmI system. Also very strong is the need to recognize an approaching person for safety reasons.

In connection with a blind person staying in a building, the need for navigating inside the building is also mentioned by respondents—see also topic papers [5,17]. However, this only concerns public spaces and not home environment, with which a blind person is indeed familiar. That is why this need was not mentioned by the blind in connection with staying at home, but only away from home.

#### 4.1.2. Blind Parents

Apart from the needs mentioned above, there is a stronger need for supervising a small child, who might do something dangerous that could not be identified by hearing. If the child is sighted, then the need for assistance is very strong. All the deficiencies of the aids or the home AmI system may affect the child, which is something that ought to be minimized. Studying or working with a text requires an input and output that would be suitable for blind parents as well as for their sighted child—e.g., the child types on a keyboard and uses a monitor, while the parent checks what the child has written by using the feedback of synthesized speech.

#### 4.1.3. Blind Child and Sighted Parents

The main goal of sighted parents should be, according to the respondents, to encourage their blind child to be self-reliant. It means that it is necessary to teach the child quite early to use certain tools and aids or to use a home AmI system for learning and taking care of themselves.

A home AmI system should focus on the child’s development in terms of the educational process. The child’s sighted parent play an important role in this process, and the AmI system should therefore support the needs of sighted parents in their interaction with their blind child (e.g., checking homework on a computer using the AmI system’s user interface for sighted and blind users). This makes it possible to encourage the blind child to be independent: in obtaining and reading learning materials; in writing documents; in using household appliances; in playing and in their hobbies; in orientation in the environment (weather forecast). According to the people who were interviewed, if the parents do everything for the child, it may have very adverse effects on the child (in terms of his or her future independence).

#### 4.1.4. One Person in a Couple Is Blind

As with a blind child, it is appropriate that a blind partner wants to and knows how to best complement their sighted partner. The aids and/or the home AmI system which they use should be a great help in this respect.

A significant concern when deciding whether to marry a blind partner is the fear that it will require excessive care for that partner, or carrying out duties that he or she would otherwise perform. A blind partner should be as independent as possible in routine household or family-related tasks (e.g., childcare). This requires the blind partner to be independent in the following task in particular:

- Being able to prepare for work or work at home (highly specific for each blind person),
- Being able to determine the identity of incoming visitors,
- Being able to obtain information of a specialist, encyclopaedic or free-time nature (e.g., reading audio books or electronic books and documents by means of speech synthesis),
- Being technically independent in the use or maintenance of appliances (basic operation of small appliances, such as kettle, microwave oven, kitchen scales, radio, vacuum cleaner; basic operation of larger appliances, such as washing machine and stove; operating the heating in the flat),
- Being independent and able to help with childcare and upbringing and with their preparation for school,
- Being able to orientate himself or herself in the environment (e.g., what is the weather going to be like in an hour? How to dress the child accordingly?).

Apart from the above, a blind person can also have specific requirements related to other potential handicaps. AmI systems using modular architecture should be particularly capable of offering the installation of other devices according to a person’s specific requirements in order to provide for the person’s vital everyday needs.

### 4.2. The Limitations of Using New Technological Products Based on User Interface

The topic of the perceived needs of blind people in home environment is closely connected to the current technological development of ICT and mainly the changes on the level of user interface. In connection with the topic of the user interface of technological products, it should be mentioned that their development over time from the perspective of use by blind people has been rather negative.

The current trend is to equip appliances with different sorts of touch control (e.g., washing machine, mobile phone, stove), which a blind person may not at all be able to operate. In mobile phones with iOS and Android operating systems this problem is dealt with by the integrated functions VoiceOver or TalkBack, respectively, which read aloud the text of a button that is being pointed to in a menu, and the respective function is activated only after a fast double-click. The problem in this case are software applications that do not support this function for visually impaired people. To give an example, the button “Send” is interpreted for the blind person in the following way: instead of the word “Send”, which is graphically displayed on the button (it can be an image button), it reads aloud the implicit labelling assigned to a given element, e.g., “Button1”. It is for this reason that the functions VoiceOver and TalkBack do not provide a blind person the expected support that would be universal and could be used in all software applications.

According to the study [78] conducted in Japan, visually impaired people with residual sight welcome the use of the touch screens of tablets and mobile phones, as their functions are in this way often ready for use by visually impaired people and they can be used for example as a reading aid. On the other hand, this study also points out that a significant number of blind people would prefer a traditional mobile phone with hardware buttons. Blind people prefer hardware keyboards, which give them feedback on (not) pressing a button. Touch screen controls fail to inspire confidence due to the problematic feedback and researchers are seeking new solutions, such as “talking touch” views for Android [79].

With the exception of devices equipped with operating systems with feedback functions for visually impaired and blind people (mainly computers, tablets and mobile phones), in most other cases a blind person is unable to use an appliance with a touch screen because there is neither tactile nor auditory feedback.

Another unpleasant obstacle appears when using a keyboard as the interface for writing and using a computer, as it was found that most available types of ordinary keyboards do not have suitable hardware. Apart from ordinary keyboards a blind person may use a special Braille keyboard and write directly in Braille script. Another option is to use a normal keyboard and touch-type as a sighted person would, or a keyboard in the so-called Braille mode can be used as long as it is supported by the software. In this case, it is necessary to press multiple keys in order to write a single character. The Key RollOver (KRO-n) parameter of a keyboard defines how many keys can be pressed at the same time. Computer Braille requires KRO-8, but most keyboards are in the range from KRO-3 to KRO-6.

To humanize informatics, it would be suitable to establish a new standard for keyboards. They could have a hardware switch or an option in BIOS that would enable KRO-8 for working in Braille. Such a systematic step could support blind people’s literacy in Braille, which is very important for them. Appendix A introduces Braille script for the blind with 6-dot as well as 8-dot characters used for the unique characters in various languages.

The topic of blind people’s literacy in Braille and its importance in the English-speaking world is discussed in detail in the article [80]. Despite the implementation of new technologies (that enable e.g., converting text to speech and vice versa), it is still important to maintain and develop blind people’s literacy in Braille—e.g., to be able to identify medicaments from the writing in Braille. This skill should be also supported by new technologies and systems, if possible. For this reason, the designed AmI system supports input from an ordinary keyboard in both modes—standard as well as Braille. In RUDO, this problem is also connected with the fact that the keyboard is the input interface and at the same time a limiting factor (low KRO for writing in Braille), mainly in working with text editors.

### 4.3. The Needs of Blind People and How They Are Met in RUDO

The design of RUDO focuses on creating such an environment that would improve the conditions of a blind person’s domestic life with a view to making easier certain everyday situations (e.g., the arrival of an unknown person, supervising children, using a computer). With regard to the everyday activities of a blind person in home environment it should be emphasized that a blind person uses a number of aids that make some activities easier for them and they are accustomed to using these aids. That is why the goal of the designed system should not be to interfere with these habitual procedures and to suddenly replace them with a completely new and complex system. On the contrary, the aim should be to retain as many familiar aids as possible and to add to them new functions. The authors believe that a sudden shift from the familiar procedures could disturb the feeling of safety and security and bring about a resistance to the implemented system, which would lead to a refusal of the system.

For this reason, the designed system RUDO focuses on providing support in those areas in which the most widely used aids (available in the Czech Republic and Slovakia) do not provide much assistance. The principal designer of the RUDO system, being blind himself, identified a basic set of aids that are used at home:

- Talking digital notebook with refreshable Braille display
- Computer with specialized software
- Level indicator (placed to the edge of a cup, a beep indicates when the blind person should stop pouring so that the cup does not overflow)
- Talking or foldable tactile measuring tape
- Talking body thermometer
- Talking room thermometer
- Talking personal scales
- Talking kitchen scales
- Talking colour indicator (placed e.g., on fabric, it recognizes its colour, on a banknote it recognizes its value)
- Talking blood pressure gauge and pulse meter
- MP3 player (regular, but selected so that it could be operated using touch only)
- Mobile phone with specialized software
- Talking watch
- Talking clock
- Mechanical typewriter for blind people (uses thicker paper and Braille script)
- Braille embosser
- Light source indicator
- Tool for banknote and coin recognition

The RUDO system is also a complement of these aids, which increases the chance of it being positively received by blind users, because it does not force the users to make any significant changes in their routines or the aids to which they are accustomed.

Section 4.1 introduced the needs perceived by blind people at home. Apart from the use of various compensatory aids, emphasis was also put on the need for a security system that would be able to identify an approaching person, the need for audio feedback from and regulation of appliances and other home systems, and the need for supervising children. Prior to developing a new (fourth) version of RUDO system, the following targets were set that concern assisting blind people: (1) assistance in recognizing approaching people, (2a) assistance in performing specialized work with a computer, (2b) assistance in studying for school, creating documents and providing information saturation, (3) assistance with parents’ supervision of children, (4a) assistance with the operation of a heating system, (4b) automatization of energy saving. Table 1 introduces the individual areas in greater detail by giving examples from a blind person’s life.

These assistance requirements were determined by the principal designer using the above mentioned exploratory research, and also by the planned installation of an AmI system prototype in a detached house (specific needs of the family). The goals (1) and (2b) in particular are also confirmed as important needs by respondents in the exploratory research. They can therefore be considered typical for a blind person’s stay in their household. The other targets are related to the specific context in which RUDO was to be implemented. Nevertheless, there might be other blind users with similar requirements. Target (2a) is connected with the work of a blind person. Target (3) is related to the parental role that some blind people have to fulfil. (4a) is connected with saving finances, since blind people tend to have very low income or are entirely dependent on social benefits from the state; and (4b) is connected to increasing comfort when at home. It must be emphasized that it is not only the needs created by the blind person’s handicap that should be taken into account here; the system should be considered as affecting the social context of the entire household, as the sighted household members are also affected by the use of the system or use some of its functions themselves. The following section describes in detail the RUDO system that focuses on those six areas of assisting blind people in home environment.

## 5. Design and Description of RUDO System

This section begins by briefly introducing the history of the development of RUDO system, which began in the year 2000. The impulse for developing it was a blind person’s need to distinguish among the people who arrive between those who are familiar and those who are unknown to that person. The first version therefore included a taxonomical system with motion detectors that worked on the principle of evaluation using a feedforward neural network with adaptive mechanism Back Propagation. This solution was published in the article [81].

It became evident that such a home assistant is very interesting and can also be used by a blind person in other areas. The second version added to RUDO a Metex digital multimeter via an RS232 serial interface for work in the areas of professional informatics and electronics. Through a home speaker RUDO reads out by means of an announcement system the electrical quantities, ranges and the values that were being measured.

The first two versions ran on a 32-bit FreeDOS operating system platform. The third version started to be developed in 2005 and had its own text-to-speech synthesizer for OS Linux Debian. The third version was completed in 2010; it operated under OS Linux, had an added speech synthesizer (called GOBLIN) version 1.0, text-processing software for blind people, and a keyboard driver that enabled writing in Braille. Descriptions of the first three versions of RUDO have not been published as a whole, and only selected parts were published in Slovak—see e.g., [82,83,84,85].

RUDO proved to be a great help, and the development of home automation systems opened new possibilities. The development of the fourth version of RUDO was completed in 2016 and currently it also includes automated heating, zone regulation and more advanced software support for blind people. This new version was briefly introduced in the Slovak article [86]. The new computer hardware used in the prototype does not presently include an RS232 serial interface and for that reason the multimeter is not yet available in this version.

Section 5.1 introduces the main hardware components and software services and describes the designed home AmI system RUDO in version 4.0 as a whole. A description of the user interface of RUDO system for blind people is given in Section 5.2. Section 5.3 introduces the main applications used by the ROWS module for working on a computer. Compared to the earlier versions of RUDO, the chief improvement is a two-phase regulation of heating that can be operated by blind users, as described in Section 5.4. Section 5.5 provides an answer to the research question from Section 1.

### 5.1. The Architecture of RUDO System

RUDO system is a network-oriented product that runs within a local computer network, and is implemented on OS Linux Debian. It includes the basic groups (or modules) of hardware components and software services listed below. The description given here corresponds to the prototype of RUDO system version 4.0, which has been actively used by a blind person since 2014. During the testing from 2014 to 2016, minor adjustments and improvements were made to this version of RUDO based on the feedback. The prototype of the system has been implemented in a two-storey detached house with a garden—see Section 6 focused on the testing period.

RUDO system and its component parts use several technology standards (e.g., protocols, specifications, models). These standards enable the integration of further components and scalability through standardized protocols and data models [35]. An overview of current standards used in technology related to AAL can be found in [87]. Standards used by RUDO system are given in Table 2.

System security is, for the time being, dealt with only on some levels. Communication protection for the remote control of RUDO is ensured by HTTPS protocol. In addition, character control strings are encrypted with a cipher that requires the knowledge of a specific encryption key. The local network, used by the individual parts of RUDO system for communication, is secured by a firewall router, which offers a wide range of services for this purpose. The Home Miniserver is protected by the standard means offered by OS Linux Debian. Communication within the local network is not secured by encryption for the moment, but the internal communication protocols are designed exclusively for this system and therefore it has not yet been necessary to encrypt them.

#### 5.1.1. Hardware Components

This section describes the basic hardware components of RUDO system that are used by the respective software services. The fact that the RUDO prototype was implemented in a family with a small child created a particular need for the supervision of the sighted child in the garden by the blind parent. The designed solution providing child supervision outside the house as part of RUDO system is therefore discussed on its own in Section 5.1.3. It should be taken into account that this is a special requirement given by the social context in which the AmI system was to be installed.

**Figure 1:**
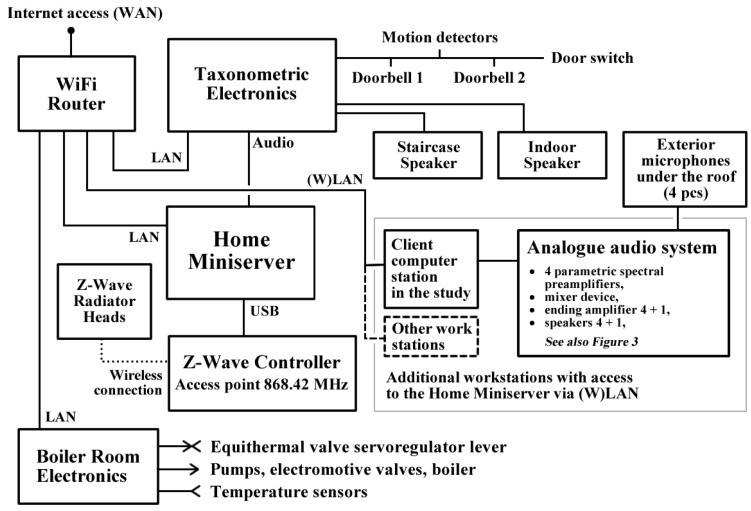
Block diagram of the main hardware components of RUDO system. This is the form in which the prototype of the system has been implemented in a detached house. Source: Authors.

The central hardware component is a Home Miniserver with which all the other system components (or modules) communicate via a router, or directly via USB. OS Linux Debian is installed on this computer. The computer is also equipped with a sound device compatible with ALSA driver for OS Linux Debian. The Home Miniserver can be operated by a sighted as well as a blind person via remote control from client computer stations with the standard equipment of keyboard and screen. The Home Miniserver does all the calculations that the software services of RUDO offer. Part of the services is also a minimizing of Back Propagation process which serves for the adaptation of a neural network for taxonomic identification. For this reason, a lower performance of this computer is not recommended. The prototype of RUDO system uses a computer with the following parameters: AMD Turion II Neo N54 dual core 2.2 GHz CPU, RAM 2 GB, HDD 250 GB, 6 × USB, 1× LAN, graphics adapter. The connection of the Home Miniserver to the router and other devices via LAN, USB and audio outputs is shown in Figure 1.

The Home Miniserver is connected with a device labelled as Taxonometric Electronics. It is a hardware input/output device that connects motion detectors, door switch and doorbells to a local computer network. The electronics of this device were designed especially for the purposes of RUDO. The device has its own IP address is connected to the router via LAN (see Figure 1).

**Figure 2:**
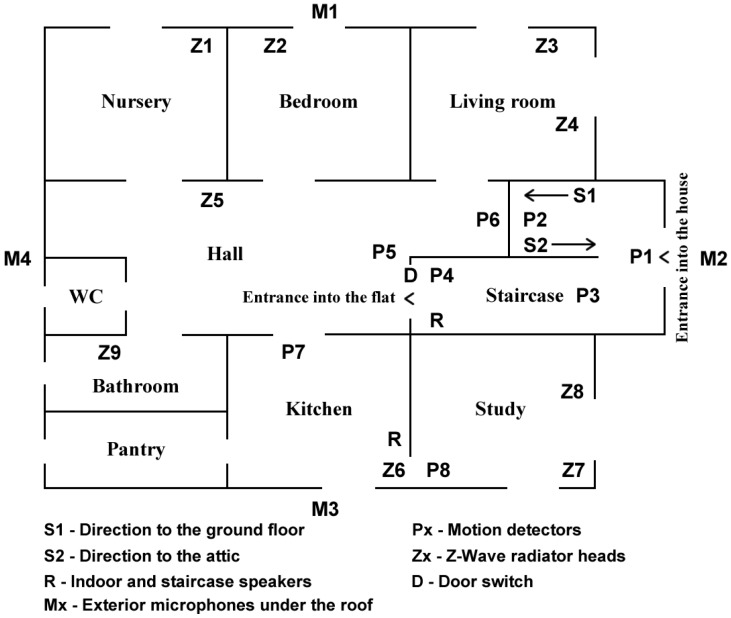
Plan of the flat in which a prototype of the system has been installed, and the placement of motion detectors and other input/output components of RUDO system. Source: Authors.

The Taxonometric Electronics also switches between loudspeakers according to the individual software services. The Software Service for Security and Taxonometry (see Section 5.1.2) communicates with this device via network programming interface MODBUS. The service input is the information collected from the motion detectors, door switch and doorbells. The service has two outputs. The first service output is a signal for switching the speakers (staircase or indoor) used for communication (see Figure 1 and Figure 2). The switch is software-controlled via LAN and MODBUS interface. This is how RUDO selects the location of a particular communication. The second output is a requirement for announcements sent to the Software Service for the Synthesis of Speech and Orientational Sounds (see Section 5.1.2). This service sends the relevant announcement or orientational sound via audio cable to Taxonometric Electronics, where the appropriate loudspeaker has been activated.

Boiler Room Electronics are connected to the Home Miniserver in a similar way. It is an input/output device that connects temperature sensors, electromotive valves, equithermal valve lever and pumps to the local computer network. The electronics of this device were designed especially for the purposes of RUDO system. The device has its own IP address and is connected to the router via LAN (see Figure 1). The Heating Software Service (see Section 5.1.2) communicates with this device via network programming interface MODBUS.

The inputs of the service are:

- data collection from temperature sensors,
- data collection from valve position.

The outputs of the service are:

- two electromotive valve controls,
- pump control,
- equithermal valve control.

Part of RUDO system is zonal temperature regulation whose hardware is provided by Z-Wave Controller and Radiator Heads. Temperature regulation in the individual zones is realized by thermostatic Z-Wave Radiator Heads. The Software Service for the Zonal Temperature Regulation, which is installed on the Home Miniserver, communicates wirelessly with the radiator heads via Z-Wave Controller that is connected via USB to the Home Miniserver (see Figure 1). Connected to the hardware input/output devices (Taxonometric Electronics, Boiler Room Electronics and Z-Wave Controller) are the end devices of RUDO system, which are:

- Eight motion detectors,
- Door switch,
- Two doorbell relays,
- Equithermal valve servoregulator lever,
- Two electromotive valves,
- Two pumps,
- Gas boiler,
- Seven temperature sensors—PT100 (part of the heating system),
- Nine thermostatic Z-Wave Radiator Heads.

The Z-Wave Radiator Heads use energy provided by batteries. The batteries last from a half to the entire heating season. Their consumption is driven by the frequency of automatic temperature corrections, so they depend on the location of a particular radiator in the building.

The transmission of the Z-Wave signal is not energetically demanding since the heads are mostly in sleep mode. The regular time when they become active is defined by the so-called wake-up interval, which is set to 5 min. This interval can be set to a maximum of 30 min, which further decreases battery consumption. Temperature corrections are always made when the head becomes active. Shorter wake-up intervals increase the speed of the head’s reaction to a user’s signal from the client application or to an automatic signal from the zonal temperature regulation system. The arrangement of Z-Wave Radiator Heads is shown in Figure 2. A detailed description of the two-phase heating regulation used in RUDO is given in Section 5.4.

#### 5.1.2. Software Services

This section introduces the main software services of RUDO system that offer a set of software functionalities. To enable working with these services, RUDO offers a unified user interface for blind people—see Section 5.2. An introduction of the ROWS module, which offers an application and script package facilitating blind people’s work with computers, is given in a section of its own, as the ROWS module can be used independently of RUDO system—see Section 5.3.

The Software Service for Security and Taxonometry evaluates data from the sensors and gives the blind person information about movement in the flat or identifies a person entering the flat. RUDO system offers two types of acoustic information connected to movement in the flat and security when someone enters the flat: orientational sounds of the environment (e.g., when someone enters the kitchen, there is a short sound of spilling water) and announcing an identified person entering the flat from outside (e.g., announcement “The person approaching is your daughter”). For these announcements, it uses the Software Service for the Synthesis of Speech and Orientational Sounds described below.

First, the orientational sounds of the environment, generated on the basis of movement in the flat, will be introduced in greater detail. The Software Service for the Synthesis of Speech and Orientational Sounds (GOBLIN speech synthesizer), which is introduced below, contains a database of sounds that are used by the Software Service for Security and Taxonometry as informational sounds. The database is relatively extensive.

The orientational sounds of the environment are created on the basis of the evaluation of data from the motion detectors and the door switch—see Figure 2. There is always a short sound (camera click, splash, etc.) when somebody appears in a particular room or when they move somewhere else. For example:

- When someone approaches by the staircase from the first floor and comes to the door between the staircase and the hall, there is the sound of a knock on the door.
- When the person begins to open them, there is the sound of creaking door.
- When someone forgets to close the door when going out, RUDO reminds them via the Staircase Speaker.
- When someone enters the kitchen, there is a short sound of spilling water.

It should be added that the sounds related to movement in the hall and entering the kitchen or the study are not always activated, unlike all the other orientational sounds of the environment. First there has to be a period of several minutes with no movement in that area, and only then the orientational sound is activated when someone moves. During normal activity in the flat there are no sounds. When everyone is moving about in the flat, they know about each other and do not have to be informed. But after a period of no movement it becomes interesting if someone comes in.

A specific informational sound announces in the Staircase Speaker that the person who is leaving has not closed the door. This event is also announced by the respective sounds in the Indoor Speaker. If the door remains open longer, the announcement in the staircase is repeated at a 5-min interval. The Indoor Speaker also repeats the respective sound at the same interval.

The second important function of this service in terms of the blind person’s security is the notification about an identified person coming to the flat. The system recognizes the familiar people who arrive and identifies unknown people by the motion detectors. Taxonometry and recognition is carried out by a neural network. Previous versions used a modification of the older technology of Back Propagation which tended to get stuck in local extremes and it was necessary to repeat the minimization process. Now, there has been developed a new adaptive mechanism based on genetic algorithms and somatic adaptive mechanisms of individual neurons [88]. This new adaptive module is being implemented into the RUDO system prototype.

The feedforward neural network with back propagation adaptive mechanism uses the installed motion detectors that give the system signals to create timestamps in the respective areas. The motion detectors are installed in a way that ensures a qualitatively different interior environment between each two detectors. That enables measuring the length of a person’s movement when overcoming a particular obstacle in the environment. The movement of a person across a scanned trajectory generates N+1 timestamps. Time differences between the adjacent stamps create an N-dimensional vector. This vector specifies the movement of a particular person by recording the times of their movement in the qualitatively different areas. It seems obvious that the motion vectors of a child are different from those of an elderly person; but even the vectors of people of a similar age differ according to how they approach the individual areas.

After generating the motion centroids [89] for a person, it is possible to measure their distance from a newly created motion vector. If the value is within the centroid’s permitted range, the incoming person is recognized. The input and output of the neural network is generally a vector. The output is then converted to a number that specifies the index in a table of recognized persons’ names. The AmI system RUDO contains a module with a feedforward neural network whose input are motion vectors. The output is a real number to which the following rules apply:

- If the output is distant from a whole positive number ≤ delta, an index has been generated
- If an index has been generated and it is smaller than the number of names in the table, the person has been recognized
- In other cases, the system gives an impulse to adapt the neural network for a new pattern (the person has not been recognized).

The taxonometry of RUDO system uses a relatively small neural network. Despite its small range, it was sufficient for recognizing the household members and differentiating between known and unknown people. The used neural network ([81,85], [89] p. 52) had the following four layers:

1. Input layer—seven receptors
2. Inner layer—80 neurons
3. Inner layer—40 neurons
4. Output layer—one effector.

In order to ensure a blind person’s safety, it is necessary to identify people who come from outside in. A detector (P2) in the downstairs hall is activated when someone comes from the first floor, and another detector is above the entrance (P1)—see Figure 2. That makes it possible to distinguish people who come from outside, which is the most critical group. The sensor placed above the door of the study becomes activated when a person is still in front of the kitchen door (P8). So when someone comes from the staircase into the flat, they go through the kitchen and before the person enters the study RUDO announces in the indoor speaker e.g., “The person approaching is your daughter”. And when the neural network does not recognize the person, RUDO announces: “The person approaching is unknown”.

The Software Service for the Synthesis of Speech and Orientational Sounds uses two speakers for announcements—a Staircase and an Indoor Speaker (see Figure 2). The Indoor Speaker covers mainly the kitchen and the study. The Indoor Speaker plays all sounds produced by RUDO. The Staircase Speaker alerts to open front door, says goodbye and welcomes the people who arrive. In order to make these announcements, the GOBLIN speech synthesizer is also part of this software service. This synthesizer can be installed on a computer independently and used by other applications (see its use by ROWS module in Section 5.3).

RUDO announces via the Indoor Speaker various statuses of the individual parts of the home system. The status reports always include a specific informational sound (e.g., guitar chord, thunder) and the announcement itself. Some examples of announcements are:

- “Batteries in the living room, south radiator, are low”—there is a similar announcement for all radiators when the Z-Wave batteries are <10%.
- “The heating should be switched on”—when automatic heating is switched off and the room temperature drops under a critical level.
- “The fire should be stoked up”—when the heating runs on solid fuel and the boiler temperature drops under a minimum level that is calculated on the basis of heat loss function.
- “Warning. The solid fuel boiler is overheated!”—when the temperature of the solid fuel boiler rises above 90 °C.

When the heating client is on, this speaker is used for related sounds, e.g., regular status report of the equithermal valve, heating activation, etc.

Apart from the synthesis of human speech, RUDO also uses a number of orientational sounds that make easier a blind person’s orientation in the movement in the house, or are connected to what he or she is currently doing on the computer. There are two types. The first are the orientational sounds of the environment that are announced by the Indoor or Staircase Speaker, already introduced in the section concerning a Software Service for Security and Taxonometry. The second are the orientational sounds that provide fast feedback when working with a computer or using a ROWS module coming from the speaker that belongs to that particular computer station (see ROWS module for work with computer in Section 5.3). This second type of orientational sounds will be briefly presented in connection with their use in the ROWS module.

The orientational sounds of the ROWS module mainly provide feedback when working with computer, and the blind person receives feedback in the form of short emotional sounds, e.g., about whether something was done successfully or not. When working with neural networks and genetic adaptive mechanism, the blind user often does not need to read the contents of a display because the sounds express what he or she needs to know. For example, for adaptation and statistical evaluation of the speed of adaptation there can be the following sounds with the respective meanings: a child’s laughter—the speed of the minimization process is increasing; a child’s cry—the speed of adaptation is decreasing; a child in agreement—the speed is constant. ROWS module is described in greater detail in Section 5.3.

RUDO system also includes Heating Software Service, which performs the heating process, water heating in the boiler and Software Service for the Zonal Temperature Regulation, which regulates temperatures in each room via Z-Wave Controller (see Figure 1 and Section 5.4). Combining both these services accomplishes a two-phase heating regulation. During the designing of heating automatization, emphasis was put on two important needs of a blind person:

- Saving energy and thus money in the long-term. This need has an increased importance for blind people due to the fact that their handicap makes it more difficult for them to find work, and even if they do their wages are typically very low. In Slovakia, for example, more than a half of blind people depends on social benefits from the state. The blind thus often live on the verge of poverty.
- Heating that can be fully operated by a special user interface for blind people (see Section 5.2). At present, heating systems with a user interface for blind people are not manufactured—see also Section 4.2 which focuses on the limits of current user interfaces for blind people. The heating can be operated on any computer connected to the local network or via remote access to the local network. There are two clients available for operating the heating by a sighted and a blind person.

This solution of zonal temperature regulation enables a blind person to acquire information about the temperature in each room—zone and enables setting the required temperatures. An important part of this client are schedules by means of which it is possible to set the heating in zones and times so that it is turned on only in the rooms that are currently being used. That creates an illusion of full-time heating that significantly saves energy with no loss of comfort. This module of RUDO system is described in greater detail in Section 5.4.

#### 5.1.3. Device Used for Supervising Children Outside the House

Although RUDO is designed as a home AmI system for blind people, it is extended by another function that is indirectly related to the inhabited environment of a home. A prototype of the system is installed in a detached house with an adjoining garden, and the blind person who uses the system has a sighted child. This created the need to supervise the small child while playing in the garden when the blind person was inside the house. A sighted person might check up on the child by looking out of a window, but for a blind person a look outside is replaced by recorded and processed sound from the exterior microphones around the house. If there is a problem, the blind parent knows in which part of the garden the child is located and they can go there to help.

Part of the RUDO system is audiotechnology adjusted for supervising children outside the house only by hearing. The audiotechnology is also used for the amplification of computer audio output. This analogue audiosystem is not directly connected to the Home Miniserver, but to the primary computer located in the study (see Figure 2). This audio-set includes:

- Five condenser microphones
- Four parametric preamplifiers (spectral filtering),
- Mini mixing apparatus,
- Final amplifier 4+1,
- System of speakers.

**Figure 3:**
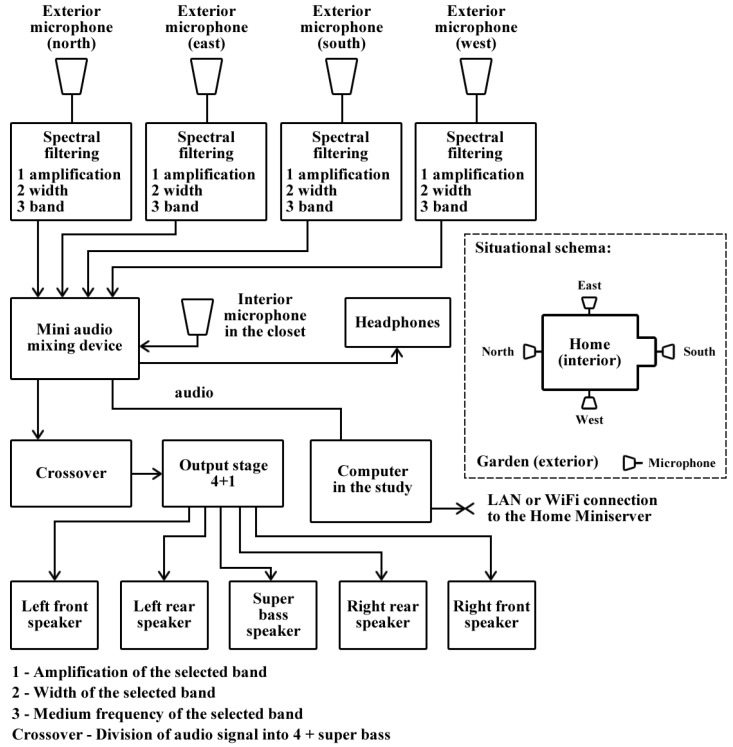
Block diagram of the Device Used for Supervising Children Outside (playing in the garden). Source: Authors.

The basis of this module are four exterior microphones placed just below the roof of the house heading in the four cardinal directions (see Figure 3). The blind person then listens to the sound of all four microphones which is processed to filter only the child’s voice. The sound from the exterior microphones is processed by parametric spectral band filter. This minimizes the interfering noises and the microphones can register children’s voices from a greater distance. The resulting sound can be heard on the 4+1 speakers in the study, as well as via headphones that do not disturb anyone else in the house. When using headphones for listening to the exterior sounds, the interior microphone in the study is also switched on, in case another household member was speaking to the blind person in the study. Thanks to this device, a blind person is able to determine the approximate location of the child in the garden and react to what he or she says, e.g., when they hit themselves and are calling for help, the blind person knows in which part of the garden the child is to be found and they can go there and help them.

### 5.2. User Interface for Blind People Used in RUDO System

The development of ICT has brought about new possibilities as well as new challenges in the area of compensating for visual handicaps. The latest development has brought products with touch displays that do not usually allow the installation of assisting technologies. Users may be faced with such barriers in the most common products such as printers, radios, DVD players, televisions, gas and electric heaters, washing machines and many others—see Section 4.2. A blind person is once again left helpless with their handicap, unable to become integrated into the current way of life.

The RUDO system communicates within the local computer network on the client–server principle. For that reason, it can be operated by client applications that are installed on the computers connected via the computer network. They can also be operated by remote access via the Internet. The client computers are equipped with assistance technologies for blind people which provide information from the client applications and by which RUDO is operated, using synthesized speech and refreshable Braille display.

The user interface is so modified that for a blind person it replaces looking at a screen. The audio output is therefore realized not only via the speakers of the client computer station, but there are two additional speakers installed in the flat that the Home Miniserver uses for announcing important events.

The following sections introduce the technologies of a special input/output user interface for blind people. RUDO uses this interface to communicate with a blind person in two ways: for personal communication with the user while working on a client computer station, and for public communication via speakers.

#### 5.2.1. Special Input for Blind People

If RUDO system is to be operated by a blind person, it is necessary to modify the input from a standard keyboard to input in Braille. The software that ensures this function reads the events on a standard keyboard from a /dev/input/eventX device [85,90], and it disables this device for other processes. At the same time, it opens the keyboard emulation device /dev/uinput [85,90] for writing.

This filtering of standard keyboard events extends its function for writing in Braille [85]. The extended keyboard function can be easily switched on and off, and therefore this modification does not inhibit sighted users.

In the extended keyboard function, the characters “asdfjkl;” are interpreted as Braille dots—see Appendix A. The keys have to be pressed together in appropriate combinations. This filtering software is installed on the client computers as well as the Home Miniserver. A blind person can therefore perform normal operations as well as overall maintenance of the home automatization system. A related problem, already pointed out in Section 4.2, is the low KRO-n of the keyboards that are manufactured—i.e., the number of keys that can be pressed together.

#### 5.2.2. Special Output for Blind People

RUDO system has an implemented GOBLIN synthesizer [85,91] that can work with the keyboard filter for blind people, with a refreshable Braille display, or it can be used for reading information. When reading information from a standard screen, GOBLIN synthesizer works with the Software Reading Assistant that assists the blind person in orientation on the screen and reading the information that is important for them at that time. It automatically selects such information from the screen that should be read with priority, and information that does not need to be read [85]. It also offers tools for imperative selection of text from the screen which is then read by synthesized speech.

GOBLIN synthesizer is installed on client computers where it uses their respective audio devices. It is also installed on the Home Miniserver. This implementation makes the announcements on Staircase and Indoor Speaker. But they can also be used for the providing of information when the client computer does not have any available audio device or when this device is used for other purposes. GOBLIN synthesizer offers several modes and speeds of speech, which also include several types of spelling that can be used for correcting written texts. The members of a household can be for example parents with a blind child who needs assistance with learning, writing homework, etc.

The GOBLIN synthesizer will now be described in greater detail. When developing the technology of synthesis for RUDO system, there were two main goals that influenced the choice of methodology and priorities. *The first goal* was that the synthesizer should be developed especially for the needs of blind computer users. That required the six points below to be fulfilled:

- Intelligible text-to-speech synthesis
- Several types of spelling
- Marking capital letters
- Different speech rates can be selected
- Orientational and emotional sounds can be generated
- Compatibility with Software Reading Assistant.

An emphasis on spelling and marking capital letters is very important for a blind person during text processing and related corrections. Speech rate setting and their skills in using the Software Reading Assistant significantly influence the overall productivity of a blind user. The orientational and emotional sounds inform the user about the events that are important for them at that moment. Specific sounds are assigned to specific events.

*The second goal* was to enable a blind amateur user to create their own speech corpus relatively quickly [85]. In practice this entails teaching a computer to speak for example in the voice of their wife. Preserving the voice of a loved one is also an interesting substitute for photographs, which a blind person cannot see.

While the development of speech synthesis was predominant for RUDO system, the speed with which the first version of GOBLIN synthesizer could be prepared was also decisive. The speech corpus for the Slovak language was therefore minimized and it contains 102 voice segments and about 100 navigation sounds. This synthesis is not of a very good quality and the synthesizer core has to include a transient generator (i.e., generator of coarticulatory transitions) because transients are not directly included in the speech corpus [85].

The second version of GOBLIN synthesizer, which is currently in development, is based on text-to-speech technology—the so-called “unit selection”. In this, every voice sample is also assigned its descriptive vector so that it can be subsequently modified; this extension was called “object selection”, because the sample and its vector constitute an object.

In order to minimize the speech corpus, texts are divided during synthesis into so-called low-level, medium-level and high-level synthesis. In texts with low-level synthesis, coarticulations are suppressed. In texts with medium-level synthesis, coarticulations are predominant. In texts with high-level synthesis, micro-coarticulatory phenomena are dominant [91].

According to the type of synthesis, the sound is composed either of phonemes and the transients are artificially generated, or it uses diphones, or triphones in high-level synthesis. The speech corpus designed for Slovak has over 500 speech objects. The automatic speech corpus generator runs through individual samples and assigns them suitable descriptive vectors. It uses spectrum and cepstrum sample analysis.

Since the speech corpus is generated automatically, the time in which it is generated is reduced to just a few minutes. The user—amateur—records through a microphone about three A4 pages of specially prepared text, which takes about one and a half hour. This second version of GOBLIN synthesizer is under development. However, the empirical testing of a partial version of the speech corpus is very satisfactory. Its quality is somewhere between the quality of diphone and triphone synthesis.

### 5.3. ROWS Module for Work with Computer

ROWS is a software module that can be used independently of RUDO system and installed on any computer. That is the reason why it is introduced in a separate section. The computer stations used by blind users, and the Home Miniserver have a set of software services installed on them, which—together with several client and special applications—comprise the ROWS module. In this module are implemented assistance technologies that help a blind person not only during standard computer operations (e.g., writing and editing of text), but also during specialist work in the field of informatics.

ROWS module uses a unified user interface for blind people, described in Section 5.2, that facilitates work with software applications on a computer. The GOBLIN speech synthesizer, that uses RUDO system, can be installed separately on client computer stations to produce synthesized speech and informational sounds via their own audio device. In the ROWS module, GOBLIN synthesizer is used mainly for reading text displayed on a standard screen. During reading it is necessary to prioritize the texts that are to be read (e.g., menus or other texts given under menus). ROWS module therefore implicitly used Software reading Assistant. ROWS module offers applications or functionalities which can be divided into three groups:

1. Applications used during normal work with computer
2. Applications used during specialized work in the field of informatics
3. Auxiliary scripts written for OS Linux.

An important application (1) is a special text editor that the blind person uses during any text processing: writing documents, processing configuration files, creating program source codes, using semi-graphic symbols to create diagrams and drafts, or creating notes on a special notepad. Text processing is made easier for the blind person not only by a unified user interface; the editor uses the GOBLIN synthesizer and Software Reading Assistant, in order to simplify orientation in the text and to help process it. Artificial speech production can also be used separately or together with a refreshable Braille display plugged in computer.

Another special application is the possibility of inserting commands into texts in a similar way as in TeX. But in this case, the emphasis is put on the simplicity of the language, which is called WORF. To create most documents with a nice graphic format after printing, only ten commands are necessary. This is an example of WORF language for formatting an A4-sized document:

          ~a4ini
          ~k1<Heading type 1~k>
          ~txt #big heading followed by text
             Text after big heading.
          ~o1<First sub-chapter type 1~o>
             Text of the first section of the first sub-chapter.
             ~no #New section command
             Text of the second section of the first sub-chapter.
          ~o1<Second subchapter type 1~o>
             Text of the second sub-chapter.
             ~nr #New line, end of document
		

A special program for printing designs for the blind person all details of the black-and-white graphic layout of a document, so that this document meets all the common criteria. The special editor and printing program are important text processing tools not only for adult blind people but also they can be used by blind children when they are doing homework, as part of their integration into ordinary primary and secondary schools.

For specialist informatics work (2) the ROWS module includes two important applications. The first application enables a blind person to create and process a speech corpus for the new version of GOBLIN synthesizer. It is equipped with another assistance technology that enables speech data processing only by means of hearing. A substantial part of the data modifications is automatized.

The second application assists a blind person when designing the topology of neural networks and their implementation into a program unit. It includes a special definition language NeuroGen and a compiler that compensates for sight during defining by automatically checking the logic of the source text on the basis of hidden information redundancies. The syntax of NeuroGen is designed so that the user has to repeat some definition elements in different contexts. At the first sight, it may seem that such information redundancies are not in the definition. During translation, the compiler checks the compatibility of these hidden information redundancies, by which it checks the blind person even when designing more extensive neural network topologies. Part of the compilation is also a strict check of the types of data on dendrites and axons and of synapse connections.

Special scripts (3) of the ROWS module are used mainly for the simplification of standard Linux commands which often require a large number of parameters with long entries. Some of the options are pre-set for a blind person and only the most important are used as script parameters.

The above text focused on the main applications of the ROWS module which makes it easier for blind people to work on a computer. The RUDO system prototype contains the ROWS module which offers further options for working on a computer at home. This is a summary of all available functions:

- Writing texts and black-and-white printing with a good graphic design
- Programmable calculator with functions for primary and secondary schools
- Diary and notebook
- Audio recording and playback with optional audio record indexing
- Environment for specialist work in informatics focused on speech synthesis, genetic algorithms and neural networks.

### 5.4. Two-Phase Heating Regulation

The preceding sections showed how RUDO communicates with a blind user. All these tools of the user interface are applied in a way that enables a blind person to use all services of this home AmI system with full comfort. As was emphasized in previous sections, a big problem in providing thermal comfort at home is the fact that current heating systems do not have a user interface modified for blind people so that they could operate it on their own. That was also the main reason for including as part of RUDO system the following three functionalities: realization of the heating process, water heating, and zonal temperature regulation.

The first two functionalities are provided by the Heating Software Service. The last functionality is provided by the Software Service for the Zonal Temperature Regulation via Z-Wave Controller. The Heating Software Service is connected via network programming interface MODBUS to an electronic device that carries out temperature measurements and operates the equithermal valve lever, two electromotive valves and two pumps. One of the electromotive valves and one pump convey hot water from the primary circuit to the heat exchanger in the boiler. The other electromotive valve and pump convey water to the radiator circuit. A four-way equithermal valve mixes hot water from the primary circuit into the radiator circuit so that the water in the radiator has the required temperature.

First-phase regulation and temperature feedback: When heating is switched on the equithermal valve is opened to maximum. The automatic regulation starts from the maximum, which makes the heating react quickly when it is switched on. The adjustment of the equithermal valve is greater when the absolute value of the temperature difference is higher. When the required radiator temperature differs from the current temperature by less than 0.5 degree, no adjustment is made because the temperature of water in the radiators is correct.

Second-phase zonal regulation: The zonal temperature regulation is operated via USB by Z-Wave Controller on the command class level. This device creates a Z-Wave wireless network through which the Software Service for Zonal Temperature Regulation communicates with the thermostatic heads of individual radiators. From a user perspective, the living space can be divided into as many temperature zones as the rooms in the house.

The first phase of regulation (four-way equithermal mixing valve in the boiler room) supplies radiators with water that is approximately one degree warmer than necessary. That gives the regulation heads space for two-phase regulation of temperature in the sense of decreasing but also increasing the required temperature of individual radiators.

Such two-phase temperature regulation creates conditions in which radiator temperatures are different in each room without excessive oscillation. The required room temperatures can thus be in the range of about five degrees without the loss of comfort. At the same time, the uninhabited parts of a building can use maintenance heating (i.e., heating at lower temperatures), and the temperature oscillations in these radiators do not matter since they occur in uninhabited zones.

#### Zone Schedules and the Illusion of Full-Time Heating

The Software Service for Zonal Temperature Regulation offers fifteen overall schedules that can be modified by the user (via client application) according to their specific needs:

1. Standard mode—going to work on weekdays,
2. In-mode—holidays, everybody at home,
3. Out-mode—nobody at home, etc.

Part of the schedules is a holiday calendar and an algorithm for calculating the date of Easter. If a holiday falls on a weekday, there will automatically be regulation as during the weekend. One overall schedule contains weekly schedules for individual radiators. A weekly schedule contains daily schedules of the radiators in which it is possible to set different heating temperatures at different times of day. The Software Service for Zonal Temperature Regulation can copy the daily, weekly and overall schedules, as well as the schedules of individual floors. Together with recorded data history it offers a powerful tool that quickly creates an entire database of zonal regulation schedules.

Energy can be saved mainly by the right setting of schedules for individual zones. Such settings must take into account all regularities in people’s lives in the environment of a particular house or flat. That creates the illusion of full-time heating, which means that the inhabitants do not feel any discomfort. But the energy savings are significant, which can be very interesting for a blind person (and their family) in the long term.

### 5.5. Answer to the Research Question

Research realized as DSR brings both practical and theoretical advantages—see e.g., [2,72,75]. A practical benefit of the research is the designed solution of a blind person’s stay in home environment. This problem is defined by the specific needs which blind people feel at home and which should be supported by the design of an AmI system—see results of exploratory research in Section 4.1. A theoretical benefit of the research is introducing the procedure used in the design and the solutions that were chosen. This may influence future strategies and approaches to solving a similar category of problems, and at the same time contribute to the formulation of more general theories concerning the design of similar artefacts.

The research question is given in Section 1. The answer to the research question is also provided in this fourth section, because it presents a principled proposal for creating an AmI system for blind people which besides providing assistance in home environment also helps with various situations and roles in which blind people find themselves involved.

The designed system RUDO fulfils the specified requirements using hardware components and software services, while the principal design of the system is independent of the used technologies. The designed system thus fulfils the defined purpose and enables blind people to be more independent in performing everyday activities connected with their stay in the home environment. At the same time, RUDO contributes to the social inclusion of blind people and their self-reliance and independence.

An integral part of DSR is also the evaluation of a designed artefact. Given the fact that a fully functional prototype was implemented in a blind person’s household, it was decided to carry out an evaluation [72] (p. 138) based on ADR. How well the designed RUDO system fulfils its function from the perspectives of a blind user and the sighted household members is discussed in the following section.

## 6. Experience with Using a Prototype of RUDO System at Home

This section introduces the experience with using a prototype of RUDO system in practice. First it briefly analyses the implementation and evaluation context. The system has undergone several years of development. Individual parts of the system were developed and pilot-tested first at the Department of Computer Science, Faculty of Natural Sciences, Matej Bel University in Banská Bystrica (Slovakia). The fourth version of RUDO, which is described in this article, has been tested at home since 2014. The prototype of the system has been implemented in a two-storey detached house with a garden, divided into two separate flats of 110 m^2^. The system has been implemented in a family in which there is one blind man and the other members of the household (a woman and a child) are sighted. The blind person works as a university teacher. This creates specific requirements concerning the implementation of the prototype in this particular problem context: assistance with specialized work with a computer in informatics and electronics (e.g., programming) and supervising a child that is playing in the garden. The testing lasted for two years, mainly for the purpose of testing the impact of implementing the module for heating and zonal regulation that could be fully operated by a blind person thanks to the connection to RUDO system, as described in Section 5.4.

It should be mentioned in this context that the vast majority of disabled people have low income, which makes the possibility of saving energy a very important concern in the design of a home AmI system. In the case of blind people, a barrier was identified in the form of the user interfaces of smart heating systems, which are not designed for being operated by blind people. The RUDO system eliminates this problem and offers a special interface both for a sighted and a blind household member. The new module for heating and zonal regulation, used in the home prototype during two heating seasons, shows considerable energy savings (see Section 6.1), which in the long-term horizon leads to substantial financial savings. This can at least partially compensate for the costs of acquiring RUDO system.

The estimated financial costs of the hardware components used in the prototype installed at home are €4000 (see the description of implementation in Section 5). But it must be added that this realization uses some more expensive devices used by the author for work, which could be replaced by cheaper versions. By using cheaper versions of hardware devices, without a negative impact on the functionality of the whole system as described in this article, the costs can be reduced to €2750. This price does not include the heating boiler and water heating exchanger. An advantage of the system is the fact that it can use both the existing heating boiler and the existing water heating exchanger. It should also be taken into account that in different buildings and with different requirements the price may vary in either direction. The return on the investment into this prototype of RUDO system is—due to the financial savings for heating and water heating—only three years. With regard to the lifespan of the installed system, the modular architecture should be positively evaluated, as it enables a simple replacement of the components of the system in case of malfunction.

**Figure 4:**
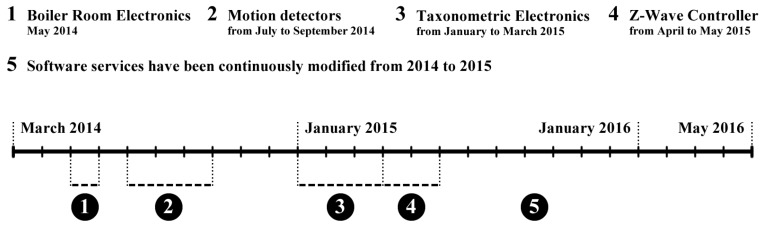
A timeline of modifications during the testing period. Source: Authors.

During the testing from 2014 to 2016, minor adjustments and improvements were made based on feedback. The timeline of these adjustments is shown in Figure 4.

Motion detectors: The testing revealed that the motion detectors react to the reflection of infrared radiation from certain surfaces of doors or glass, mainly in the summer. The reflection caused some detectors to react incorrectly. The placement of some motion detectors was therefore adjusted during the testing in order to create an architecture that would correctly measure the time differences in the movement of people (see Figure 2). Z-Wave Controller: During the design of Z-Wave, it proved impractical to have the Z-Wave heads in both flats in the house connected to the came controller (central heating was necessary in the whole house). Changes in one flat required a shutdown in the other flat as well, which was unpleasant for the inhabitants. The current version has two Z-Wave controllers in order to make changes on one floor possible without a shutdown on the other.

Boiler Room Electronics: The electronics were originally connected via WiFi, but this caused interference and failures due to the position of the boiler room. Stability during data transfer to the boiler room electronics was only eventually achieved with a direct metallic LAN connection. Taxonometric Electronics: The first version of the connection of motion detectors to the home server was realized via a USB port, to which a galvanic isolator was connected. Despite the galvanic isolation of motion detector circuits from the computer, there was interference and inaccuracy. The problem was solved by a new galvanic isolator connected directly to LAN and with its own IP address. Software services: There were many software changes during the testing period. Given their great number, they will not be detailed in the article.

Evaluation according to ADR [75] is contextually focused—see evaluation methodology in Section 3.1. In order to understand the created sociotechnical interaction, it is therefore suitable to evaluate the AmI system in context mainly from a qualitative perspective based on observation and user interviews. The feedback can be used on this basis to define the limiting conditions and to further improve the artefact within its engineering cycle in design science.

As the RUDO system is rather extensive and composed of a number of modules (components and services), the final evaluation is divided into the following two parts: technological evaluation connected with the fulfilment of the identified needs of blind people (in Section 6.1), and user evaluation from the perspective of sighted household members, or in terms of a cooperation between a blind and a sighted person (in Section 6.2 and Section 6.3). The user evaluation of the whole system is presented from the selected user perspectives: user interface, identification of approaching people, notification about movement in the flat, orientational sounds, heating and zonal regulation, and supervising children.

### 6.1. Evaluation of the Fulfilling of the Needs of Blind People

This part evaluates the home AmI system RUDO in terms of the fulfilment of the needs of blind people that were defined in Section 4.3. To make more clear the fulfilment of the individual needs by the designed system, Appendix B provides a diagram connecting needs, technological solutions (i.e., the respective module comprised of hardware components and software services), and the main functions connected with the given solution. The evaluation of the fulfilment of needs is carried out from the perspective of the technological solution of the implemented prototype. The central point of RUDO is the Home Miniserver, whose load in the individual modes was as follows:

- Without the heating system and zonal regulation, the average miniserver load was 5% at 800 MHz, which is about 1.7% in terms of the overall performance.
- With the heating system and zonal regulation, the average miniserver load was 30% at 800 MHz, which is about 10% in terms of the overall performance.
- During the training phase—the adaptation of the neural network to new patterns—the miniserver load was 100% at the frequency of 2.2 GHz.

It can be said that the load and therefore the energy demands for calculations are low, which also reduces the financial costs of the operation of the whole system. The modules of RUDO system are evaluated in terms of the individual needs in the further subsections.

#### 6.1.1. Assistance in Recognizing Approaching People

The important identified need of blind people at home—recognizing approaching people—that was fulfilled by using motion detectors and neural network, was in the course of the development of the system extended by informational sounds, which then proved very popular. Together they not only enable the recognition of approaching people, but also inform the blind person about the movement of individual household members, which brings the blind person important advantages for communication—see Section 6.2.

The person recognition uses a feedforward neural network containing seven receptors, one effector, 120 internal neurons and 3800 synapses. Such a network makes no special demands on computer hardware during recognition in active mode. The neural network was trained to recognize ten members of three households.

After the training, which lasted about half a year, the network was able to correctly recognize those people. During training, it was necessary to react to contact with an unidentified person by typing their name on the computer keyboard and initiating network adaptation to a new training pattern. The writing of the new training pattern was done by the blind user only when he was sitting by the computer—training time can therefore vary significantly. The testing period revealed two flaws in this recognition approach: (a) the motion detectors are not very precise when creating the timestamp with regard to the contact at a given trajectory point; (b) such a system does not allow full biometric screening.

A greater screening precision would reduce the network training time and increase the maximum number of recognized people. In point (a) it is possible to replace the motion detectors e.g., by laser beam, the intermission of which creates a timestamp precisely at that point of the captured trajectory.

Motion capture (timestamps from the motions detectors) does not make any special demands on the hardware. But a problem appears when information sound events are quickly generated and create a queue of sounds waiting to be processed by the computer’s audio equipment. The AmI system RUDO only has one audio device, whose output is switched between two sets of loudspeakers—indoor and staircase. The following problem with timestamp response time emerged during testing, usually during contact with the child. The running child, coming up the stairs from outside into the flat, triggered three informational sounds at first:

- A welcome on the stairs
- A knock in front of the door to the flat
- The sound of a creaking door when opening the door.

When the welcome was still being generated, the running child activated the sensors triggering two more informational sounds, then she got to the kitchen and by the time she entered the study, RUDO was just beginning to generate the knock at the door. However, it was only the small child whose movement was so quick during the testing period. This problem can be removed by installing multiple separate audio devices for the announcements in the indoor and staircase speakers.

The newly implemented and tested adaptive mechanism using a genetic algorithm accelerated the learning process from tens of hours (in the case of the formerly used Back Propagation algorithm) to just a few hours. These figures are rather approximate, as this is a random process, or in the case of Back Propagation a random initial condition. What must be taken into account at the same time is the extent of the neural network (the number of neurons, synapses) and the extent of the task (the number of training patterns). It should be added that the process of neural network adaptation runs in the background and does not interfere with the normal operation of the AmI system RUDO. It follows from the evaluation that the fulfilment of this need is satisfactory, with the weak spots of the technological solution identified.

#### 6.1.2. Assistance in Performing Specialized Work with a Computer, Assistance in Studying for School, Creating Documents and Providing Information Saturation

These two identified needs reflect the two levels of working with a computer. While the need of advanced work on a computer is related to the implementation of RUDO system prototype in the given social context (the blind person is a university teacher), the more basic work on a computer is nowadays an important basic need, mainly for the following reasons: preparing documents for school or work, social inclusion, the continuous development of information society and the related demands on its members (e.g., e-Government and the ability to communicate electronically with the public administration).

Important for these purposes are mainly two fields connected with the writing and text editing of documents: the Braille driver for an ordinary keyboard and the use of the WORF language. The text processing function enables a blind person to be independent in creating and editing texts. It enables fast and effective writing in Braille script, so the blind person can cooperate with sighted colleagues without limiting them by his or her lower productivity.

The introduced method of processing texts has one technical deficiency connected with the conversion of documents from WORF to MS Word format and vice versa. When a sighted co-worker adjusts e.g., the format of a document or adds a comment, the change may not be registered, mainly when converting the document back to WORF.

This drawback was compensated for by an agreement between the sighted and blind colleagues concerning the modification of texts. In spite of the mentioned drawback, the blind academic has been able to successfully create—with the help of the tested system—not only qualification works but also the manuscripts of four monographs. In the field of programming, this system was used for the writing and debugging program source codes in the extent of about 150,000 program lines. RUDO system offers environment for specialist work focused on natural language processing, genetic algorithms and neural networks, which was used in the development of this system. It follows from the overall evaluation that the assistance for working with a computer was satisfactory.

#### 6.1.3. Assistance with Parents’ Supervision of Children

The installed prototype distinguishes between child supervision inside and outside. Inside, there are orientational sounds of the environment, which are generated on the basis of movement in the flat. This gave the blind person a good idea of other people’s movement around the flat. The supervision of the child outside was aided by a specially created device.

This audiosystem for monitoring small children outside is considered to be the weakest element in the designed prototype. As long as the child was playing within the distance of ca 20 m from the house and made sounds, it was possible to localize her. But when the radius was greater or when the child was silent, the localization was incomplete.

With the above mentioned exterior layout, and considering the nature of the supervised child, the localization success rate was as follows: precise localization 30%, sufficient approximate localization 30%, insufficient or no localization 40%. The fulfilment of this need is satisfactory, but the technological solution has significant failings.

#### 6.1.4. Assistance with the Operation of a Heating System and Automatization of Energy Saving

These two needs can be again divided into the basic (enabling a blind user to operate the heating system) and the advanced level (providing greater heating comfort and energy savings that lead to reducing the costs). The operability of the heating system and zonal regulation must be evaluated at three levels: (a) normal user operation, (b) administrator operation, (c) operation during malfunction.

During the two years of testing, the respective module of the RUDO system prototype did not require any further assistance from a sighted person in points (a) and (b). There are several solutions designed in the system for the cases of malfunction, which a blind user can use to replace the computer automaton. However, during the two years of testing there only occurred a single malfunction and it was not connected with the heating software or heating electronics. It was the router that stopped working, which disconnected the boiler room equipment from the software automaton on the server.

Heating with this malfunction involved greater temperature fluctuations and oscillations on individual radiators. In the long term, this malfunction would result with increased power consumption. After being notified about the problem, a technician was called who repaired the router and the heating system then resumed normal operation. In the case of a more serious malfunction, even sighted people tend to call a technician, and therefore it can be said that in point (c) the system offers a comfort equal to that of a sighted person.

From user perspective, this module offers more than just significant energy savings (see Section 6.2). An important point is the complete independence of the blind person in operating the heating system. This need is fulfilled excellently.

### 6.2. Evaluation from a Blind User’s Perspective

*Identification of approaching people and notification about movement in the flat*: It was not the goal of the person recognition function to acquire full biometric data. It was only necessary to recognize a small number of known people and to distinguish them from those who are unknown. RUDO fulfils this goal. But the testing showed that there is still a need for a full biometric recognition of people coming from outside. Such a system requires a camera that would capture the space before the front door, and a recognition software. However, this module combined with orientational sounds fulfils the required purpose.

*Orientational sounds*: The orientational sounds were originally added to RUDO only as an embellishment. But further testing showed that blind people react to these sounds spontaneously in the sense of conditioned reflexes. Without becoming diverted and having to interrupt their work, they can be informed about which zones are currently inhabited. A blind person can start to communicate with other household members in different rooms without them first having to announce that they have come for example from the ground floor, from outside or from another room.

The orientational sounds, together with the recognition of approaching people, became a very useful means for a blind person to start communication, and created a higher standard in becoming aware of the presence of other household members. For that reason, the orientational sound module was first revised with great accuracy so that it would be sufficiently informative but at the same time would not intrude by repeating the orientational sounds too frequently.

*User interface*: An important feature of the special interface for blind people in RUDO is its orientation on the text console. It is therefore not necessary for the blind user to become familiar with the graphical environment of the user interface. Graphical environments tend to be relatively complex, and orientation by means of speech synthesis or refreshable Braille display can be rather lengthy. Entering short commands directly in the command line, or launching semi-graphical environments considerably speeds up work on a computer.

A drawback of this solution is the fact that the blind person is required to memorize several dozen short commands, but experience has shown that the user can learn to use these commands in a matter of several months of working with the system, even without having to laboriously memorize them at the beginning. A “help” command is available, in which the user can find a list of command for simple text searches.

Given the varied requirements of blind users, it would be suitable to extend the user interface of this AmI system by the option of operating it in graphical environment for the benefit of sighted household members. There is such an option in RUDO, but it is limited to operating the heating system and zonal regulation.

A very important feature of RUDO is providing to a blind user a complete access to heating system operation, both in standard and technical mode. Moreover, RUDO system communicates with a blind resident by means of speakers and speech synthesis, which can replace a look at the screen, or it can alert them to a radiator head battery running low. This makes the blind person completely self-reliant in this area and not dependent on the help of sighted household members.

Texts or commands can be written by a blind user either by standard ten-finger typing, or by using a Braille driver that redefines a standard keyboard to Braille mode (see Appendix A). In Braille mode, characters are written in the Braille script for blind people. The Braille driver in RUDO was tested by a blind person who was further disadvantaged by a slight paralysis of the right hand. This prevented him from using standard ten-finger typing. The Braille mode of the keyboard turned out to be the only solution that provided sufficient writing speed that would enable the blind person to be fully employed and competitive in the labour market.

Despite the extensive handicap, he achieves the following speed of writing texts and commands, using a standard non-Braille keyboard with the Braille driver that is part of RUDO:

- Minimum speed 203 characters per minute +3 corrections
- Maximum speed 234 characters per minute +0 corrections
- Average speed 221 characters per minute +1 correction.

During the testing, the computer pronounced (using speech synthesis) the characters that were pressed, on the basis of which the blind person was able to correct typing errors at once. In faster typing, the spelling feedback shortens the sounds to the minimum length that is required in order to preserve intelligibility.

RUDO system supports the use of a refreshable Braille display as well as the use of speech synthesis. Synthesis is used mainly for reading texts, and the refreshable Braille display for editing texts or programming. Reading programming code and checking grammar is much faster when using a refreshable Braille display. For example the symbol “]” can be immediately identified by touch, but in speech synthesis it is described as “right square bracket”, which is rather time-consuming. Prolonged reading time decreases a blind person’s competitiveness in labour market.

A frequently used feature of the user interface is the system of using semi-graphic symbols which makes it possible to create diagrams and drafts. All diagrams and drafts in this article were originally created by the blind author and then converted to images by the co-author. RUDO thus offers blind people another means of expression that would be very difficult to achieve in text mode.

The user interface using Braille driver and speech synthesis feedback for checking turned out to be a powerful tool in text-based communication, e.g., e-mail. The ROWS module used in RUDO makes it more natural for blind users to operate a computer via command line and offers a number of supporting tools (see Section 5.3). It is also sufficient for use at work, where this module is also installed. ROWS module and GOBLIN synthesizer enables performing specialist and administrative tasks both at work and at home. That gives a blind person better chances to succeed on the labour market.

It should be added that the components of ROWS module were also installed individually to other disabled people, or specifically modified to suit their needs. One of them was a person suffering from infantile paralysis and the movement of his hands and arms was significantly limited. The keyboard driver that was created divided keys into groups. A group of keys constituted one virtual function block which this person was able to hit despite the fact that his hands trembled considerably. It can be said that the ROWS module offers sufficient variability that makes it possible to adjust the user interface to the specific needs of a blind person who has another handicap.

*Supervising children:* The child supervision audiosystem in RUDO has a supplementary character. Despite the fact that for the blind parent it was a very important educational and protective tool during childcare, there are many flaws.

In the first place, the recorded sound should be digitalized and further processed by a computer. Digital processing achieves better results in spectral filtration and thus a higher intelligibility of the resulting sound, as well as lower degree of interference from other sounds. Child supervision would also require as part of the indoor system a taxonometric module equipped with motion detectors and cameras. Recognition software could in this area mean a dramatic change in compensating sight and preventing accidents. But in spite of the described limitations, this module fulfilled its basic function, at least outside the house in the garden where the prototype is implemented.

*Heating and zonal regulation*: Before the implementation of RUDO in the version described in this article, there was an older system of central gas heating in the building. The building had vacuum windows and heat insulation only in the roof. After RUDO was implemented and the old central heating was connected to its control unit, together with introducing zonal regulation, the savings of energy reached 40% in a heating season. During the testing of the prototype, the shortest possible wake-up interval was selected for the purposes of testing empirically the maximum consumption. Savings are achieved in two areas:

- Automatized control of heating and water heating
- The illusion of full-time heating created by zonal regulation.

If the residents do not have a regular schedule, such as going to work, school, etc., the illusion of full-time heating cannot be effectively created. A drawback of RUDO system in this field is its inability to automatically adjust the heating of the zones. It is based on an automatized statistical evaluation of zone (room) inhabitation and a subsequent change of the schedules of individual radiator heads. This function would require the installation of motion detectors in all rooms of the intelligent building, which the introduced prototype does not yet include. The main advantage for a blind person is their self-reliance in operating the heating and an environmentally-friendly approach to using energy.

### 6.3. Evaluation from the Perspective of Sighted Household Members

*Recognizing approaching people*, *notification about movement in the flat*, *and orientational sounds*: The modules described above were designed solely for use by blind people. Experience showed that also the sighted household members soon became accustomed to the informational sounds and started to perceive them in connection with the events that had triggered them. It turned out that the system of informational sounds made movement in the flat more transparent, which increased the comfort of living both for the blind person and for the sighted household members. The sighted household members also got used to the sounds and accepted them as above-standard comfort. On one occasion when the orientational sound module stopped working, it was the sighted household members who expressed the greatest displeasure.

*User interface*: When a blind person cooperates with someone sighted it is very important that sighted people do not have any contact with assistance technologies and are able to see their joint work on a screen in the usual way. RUDO therefore “hides” the assistance technologies from the sighted people. For example, a blind person types in Braille, but the operating system already receives standard keyboard events, i.e., standard characters. The blind person thus types in Braille and the sighted person reads it on the screen in the Roman alphabet.

Vice versa, the sighted person can type on a standard keyboard and the blind person reads it in Braille on a refreshable Braille display or listens to it in synthesized speech. By this approach RUDO made it possible to communicate in the Roman alphabet when working with texts together or helping a child to study. An important aspect is the possibility of black-and-white printing, which helps for example with one-way correspondence of a blind person to a sighted person, or with homework done by blind children.

*Child supervision*: A sighted person would have no need for the child supervision audiosystem.

*Heating and zonal regulation*: It is very important for the heating system and zonal regulation that these systems can also be operated by sighted people without coming into contact with assistance technologies for blind people. RUDO therefore includes software clients for operating heating and zonal regulation that work in the standard graphic environment. The heating and zonal regulation can thus be operated from standard computer stations.

A possible extension for sighted household members of the client for operating heating and zonal regulation is porting it from the desktop to a mobile device such as a tablet. The tablet can be easily attached to a wall and run nonstop and thus directly influence the heating process. But this need of sighted people can be considered a luxury and is not that important. Moreover, creating clients for tablets is technologically possible with no need of extensive development. This module is connected to energy savings, which are important both for the blind and the sighted household members. Given the increasing prices of energy, this is very welcome and desirable also from the ecological perspective.

### 6.4. Limitations of the Current Prototype and Intended Further Development

When summarizing the observations concerning the functionality of the home AmI system prototype, the following could be considered the main strong points:

- The user interface for blind people, and the support of working with computer
- Notifications about movement in the flat, and informational sounds
- Accessibility and effectivity of heating and zonal regulation.

A weaker point is the child supervision audiosystem. But this was not such a burden for the blind person in this case because he lives in a family of sighted people. The limitations of using RUDO for assistance during work with a computer can be seen mainly in the constraints given by keyboards with low KRO-n (see Section 4.2). The hardware of most keyboards is unsuitable for use in the Braille mode offered by the ROWS module in RUDO.

Among other limitations of the current RUDO system prototype is the fact that it is the only functional prototype and all the functions were not tested by a greater number of blind people. Only some parts, such as the ROWS module, were used for creating specific systems for other disabled people. This limitation is also a logical consequence of the fact that RUDO always has to be implemented in the domestic environment of a particular blind person. But with a different user of the system, the environment (the layout of the flat) also changes, as well as the context in which the system is implemented. This can be for example a blind person living with a pet (e.g., a dog or a cat), which the original design of the system does not take into consideration and it would require further modifications or module variations of RUDO.

During the development of the components of RUDO system by the blind person it was very helpful that a multimeter was connected in the second version, as well as the ROWS module which made it possible for the blind person to do work in the field of specialist informatics and electronics. These modules fulfilled their purpose and were very helpful during the realization of the whole project. But because it is a very specific and limited area, this topic was not presented in the article in greater detail. Currently there are plans for four main improvements to RUDO:

1. *Reconnecting the multimeter*: Since most new computer hardware does not currently include serial interface RS232, an innovation in reading multimeter data is currently in development, and it is also to be extended by automated tracing of oscilloscope curve.
2. Implementing a new version of GOBLIN synthesizer that would provide a higher quality of speech synthesis.
3. *Improving the recognition of approaching people*: Further development should also include a taxonomic core solution with a new neural network with a genetic adaptive mechanism. The first version of RUDO was equipped with a neural network that provided the taxonomy and identification of approaching people. A new adaptive mechanism based on the principle of genetic algorithm, that replaces the Back Propagation adaptive mechanism, is currently being successfully tested on the prototype of RUDO system.
4. Improving the security of communication between the individual parts of the system within the local network by using encryption.

## 7. Discussion and Conclusion

The RUDO system is still in the prototype phase for the purpose of researching AmI systems for the blind. It is therefore not primarily focused on complete flexibility of installation under any household conditions, and its flexibility is still quite limited. However, RUDO offers a number of user settings that can be done during installation or during subsequent use through configuration files. The settings in configuration files are diverse and rather extensive, which is why they are not presented in the article more closely. The system is currently localized for Slovakia, where the research is conducted, but it could be localized for another language and country by changing the core of the synthesizer and the tables of the Braille script. RUDO is a network-oriented and therefore modular system and its modules can also be used independently. An example of independent use is the ROWS module, which can be easily installed on a work computer away from home. A blind user can thus use the unified user interface and the programs that he or she is used to. To illustrate the adaptability of the system, Section 6.2 describes the adjustment of RUDO’s driver that enabled writing in Braille with an ordinary keyboard to a person suffering from infantile paralysis whose movement of hands and arms was significantly limited. That is why the authors believe that this system is significant beyond the customization for the single user context that was introduced here.

The presented research is important not only with respect to defining the needs of blind people in home environments, but mainly for testing the solutions designed here to fulfil those needs. Although the research does not deal with all the possible needs that blind people may perceive (they might have other handicaps beside blindness), it provides an important contribution regarding the possibility of fulfilling the selected needs in a particular social context. Apart from increasing the independence of a blind person in their household, a substantial advantage is the improved social inclusion within the family in which the blind person lives (unless they live alone). This is achieved by sociotechnical interaction between a blind person and the AmI system, which provides him or her with information about the events that occur in the flat, and it allows a new level of social communication with other household members—information about approaching people and movement in the flat, supporting cooperation with a sighted person when working on a computer. Thanks to the provided information, the blind person becomes an equal family member and does not have to constantly requite information (e.g., Who is it? Where are you? What is happening?) from the sighted family members. Based on the research of literature, some parts of RUDO system can be considered original or unique. This fact has already been mentioned in Section 2.3 in connection with the conducted research of literature.

The blind user, who has lived since 2014 in a household in which the AmI system RUDO (version 4.0) had been implemented, started to be more independent and self-reliant in the areas of supervising small children and normal and specialized work with computer as part of his job. Thanks to the taxonomic system that detects the movement of people, and thanks to the orientational sounds, he was able to achieve full orientation in the circumstances of the movement of people within the household. He was able to fully operate the heating system as a user as well as technically, just as he would without a visual impairment. He assisted his child in learning for school and doing homework.

Eventually, the sighted household members started to consider him equal in the medical sense, so he was sometimes asked to perform such tasks that would otherwise be easier for a sighted person, e.g., “What time is it, please?”, “Where is our daughter?”, “Who is at the door?”, “Could you turn the heating on?” Such questions and requests started to be common despite the fact that he was not always able to answer the questions, which confirms the new way in which the blind person started to be accepted and his new role among sighted household members. However, the home AmI system RUDO enabled the blind person to quickly and correctly react to most of the enquiries. The higher standard of services offered by RUDO system thus caused that the blind person started to be in demand as someone capable of dealing with a number of situations and problems that had until then been difficult for him to solve (e.g., prompt e-mail communication, supervising and teaching children, heating).

A limitation of the output of this article is the fact that the evaluation of the implemented prototype was carried out in the household of RUDO’s principal designer, who is blind. On the other hand, similar methods of involving researchers in evaluation are common in the technology-oriented AR, and useful for the further development of the designed artefact—see Section 3.1.

In spite of the limitations of the current version of the system, the RUDO prototype that was constructed and tested points to an interesting direction in the development of information technologies that can create for a blind person such an environment in which he or she can not only take but also give and live a productive life. This is a new approach to the integration of blind people, because this type of AmI systems can be installed not only in domestic environments but also in schools and other public spaces. That opens the possibilities for a great number of directions and approaches which require further development and integration of such systems into the environment of intelligent buildings in general.

From a broader point of view, technologies based on the Internet of Things (IoT) offer good prospects for the future development of AAL and AmI systems in general. This trend of development is pointed out in the article [29]. In the field of AmI systems for blind people the crucial point is the user interface which the AmI system offers. Individual aids or objects could be wirelessly connected to the AmI system and could be partly or fully operated via a unified user interface—e.g., fridge settings, washing machine or oven programs. In RUDO, the appliances could announce their status via the Indoor Speaker or be operated (program setting of a given appliance) with the use of the ROWS module. The advantage would be that blind people would not have to get accustomed to different user interfaces (which are often unsuitable for blind people—see the problems mentioned in Section 4.2) with each new tool or appliance. And vice versa, the tools could use some outputs of AmI system modules as a source of further data for their operation.

Interesting in this connection are also the technologies for monitoring and localization inside buildings based on ultra-wideband (UWB)—see e.g., [92]; at present they are already affordable. This technology offers high precision and reliability of the results of localization inside buildings (30–50 cm), a considerable range within buildings (about 30 m), and enables a high density of tags monitored in a particular space (the maximum is approximately 10,000 tags per 200 m^2^). Using such technology in systems like RUDO would enable not only a better monitoring of the movement of people in the flat, but on request it could also determine the position of a thing used by a blind person. Moreover, these tags can be passive as well as active, which means that they could be used to request help or assistance, which would further increase the feeling of safety and security, mainly in elderly people.

## Figures and Tables

**Table 1:** Illustrative examples of activities connected with a particular type of assistance. Source: Authors.

No. of Target	Illustrative Examples
(1)	Somebody is entering the house or flat and the blind person is informed of it; incoming family members are recognized and announced; notification about the approach of an unknown person; acoustic information about movement in the flat thanks to which the blind person can address the people around or expect communication.
(2a)	Special applications, linking and reading values from a multimeter enables advanced work on computer in the field of informatics and electrotechnics, e.g., measuring electrotechnical quantities, programming, creating neural networks, natural language processing.
(2b)	Creating formatted text documents, semi-graphic schemata and pictures, using a programmable calculator with mathematical functions for primary and secondary school; work with both standard and Braille keyboard and using speech synthesis and informational sounds for feedback during work on a computer; cooperation between a sighted and a blind person on creating a document (e.g., checking homework).
(3)	Information about the movement of a child in the flat or in the garden around the house.
(4a)	Being able to set any value in the heating system; notifications about temperature and the heating process by means of speech synthesis (including failures).
(4b)	Being able to operate the system that maintains the illusion of full-time heating.

**Table 2:** Overview of standards used by RUDO system. Source: Authors.

Standard	Application
Z-Wave	Wireless network Z-Wave is used for data transmission between the Home Miniserver and Radiator Heads.
HTTPS	HTTPS network protocol is used for encrypted data transfer when operating the system remotely.
TCP/IP	Communication with hardware components is carried out within LAN by means of TCP/IP protocol.
MODBUS	The network program interface MODBUS is used for connecting software with the hardware network equipment of the system.
Client–server model	The software components of the system are connected within LAN by means of client-server communication.
KRO-n	KRO-n is a specification of standard keyboards—the maximum number of keys pressed at the same time. Using Braille in Slovak requires KRO-8.
Ethernet	Ethernet is used for connecting selected hardware components to LAN by twisted pair cables.
Wi-Fi	Wi-Fi is used for wireless connection of the system with mobile computer workstations.

**Figure A1:**
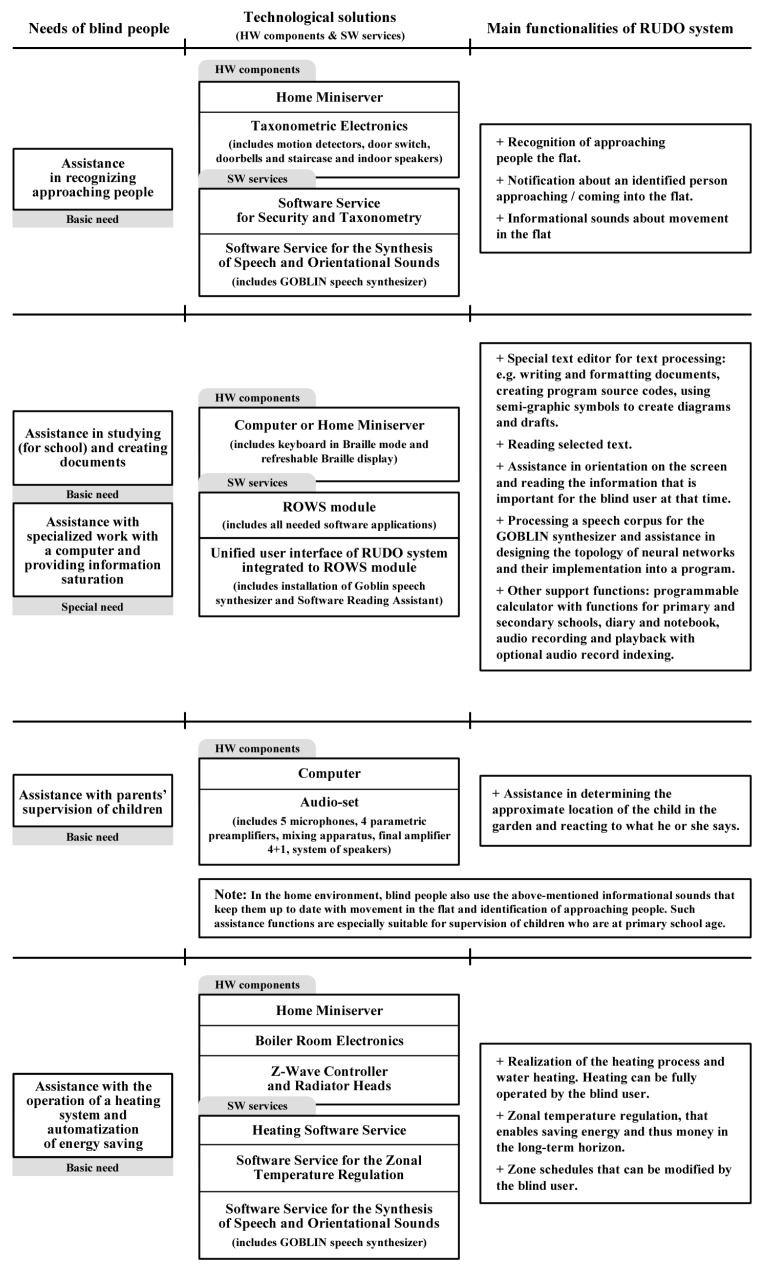
Scheme linking identified needs of blind people, technological solutions and main functionalities associated with the RUDO system.

## Abbreviations

ADR	Action design research
AR	Action research
AAL	Ambient assisted living
AmI	Ambient intelligence
ALSA	Advanced Linux sound architecture
BIOS	Basic input-output system
DSR	Design science research
GIS	Geographic information system
GB	Gigabyte
GPS	Global positioning system
HDD	Hard disk drive
HTTPS	Hypertext Transfer Protocol Secure
ICT	Information and communication technologies
KRO	Key Rollover
LAN	Local area network
OS	Operating system
RAM	Random-access memory
SmE	Smart environments
TCP/IP	Transmission Control Protocol/Internet Protocol
TAR	Technical action research
UWB	Ultra-wideband
USB	Universal serial bus
WAN	Wide area network
WLAN	Wireless local area network
WiMAX	Worldwide interoperability for microwave access

## Appendix A. Character Input Options for Work with a Text Editor

### Appendix A1. Using the Keyboard in Braille Mode in the RUDO System

The Braille keyboard mode is realized in the RUDO system on the level of the keyboard driver. This driver enables reading from the keyboard and disables it for all other processes of the system. At the same time, it opens for writing the device for software keyboard emulation. On the basis of these two actions, the operation system will read events only from the software emulation device. In other words, no other standard or special software knows whether the characters are written in the normal way, or in Braille.

*A simple example*: In the commonly used editor “Writer”, a blind person presses simultaneously dots 1, 2, 3 (see the dot position in Braille below), which are represented by simultaneously pressing the keys “fds”, and the editor receives the character “l”. In other words, working with Braille is the same as with the standard Roman script. The keyboard driver leaves all the common functional and prefix keys unchanged.

Control characters are an exception; in Braille they are represented by adding an extra dot to a given character. For example, “b” are dots 1, 2 represented by the keys “fd”. CTRL + “b” are points 1, 2, 7 represented by “fda” keys. The keyboard driver in RUDO hides from the operation system the special use of keybord. It can therefore be used without any restrictions.

In 6-dot Braille script, the three dots on the left are numbered 1, 2, 3 from top to bottom and represented by “fds” keys. The right dot column is numbered 4, 5, 6 from top to bottom and represented by “jkl” keys. In 8-dot script, there are two additional dots, one under each column. These two dots are numbered 7, 8 and represented by “a;” keys. But the operation system does not know about this, as it only receives a standard event with a so-called scancode and a standard ASCII conversion.

The Braille keyboard function is activated by pressing simultaneously LeftCTRL + RightALT, and deactivated by LeftCTRL + RightCTRL. Such form of switching the Braille function on and off does not block the standard function key combinations.

When Braille mode is on, the row of keys above and below “asdf”, “jkl;” is blocked to prevent typing errors. In addition, RUDO reads out each key that is pressed to provide feedback. This happens both in Braille and in standard keyboard mode.

When the keys are pressed in quick succession, the reading of each new key interrupts the previous. With a certain level of skills, this does not slow the writing down and the beginnings of speech sounds provide sufficient feedback.

### Appendix A2. Using the Keyboard in a Standard Way

If the driver is not installed, a blind person can use ten-finger typing like a sighted person. But this approach creates an increasingly large group of blind people who are illiterate in Braille and cannot read Braille print on paper. Braille keyboard driver can also be adjusted to a combination of handicaps. For instance, when a person is unable to fully use both hands for the ten-finger style of typing, the Braille mode used in RUDO can solve this problem and allow a speed comparable to trained ten-finger typing.

The limitations of this approach are given by the possibilities of standard keyboards. On a keyboard with KRO-n lower than 8 (necessary for Slovak, as well as other languages), some characters have to be written by pressing multiple smaller combinations. That considerably slows down the speed of typing and the opportunity for Braille literacy training is lost as well.

### Appendix A3. Using Systems for Speech-To-Text Synthesis (Speech Recognition)

Current speech-to-text conversion systems are not yet completely reliable for every voice and every language—particularly for languages that are not much used worldwide, such as Slovak or Czech. Moreover, these systems do not edit texts in a way that would make a user’s speed comparable to that of typing on a keyboard. Telling a computer to move the cursor to a particular word on a particular line and to change it to a different word is a very laborious process. A blind person is already slowed down by their handicap, and such work would make them even more disadvantaged on the labour market.

On the other hand, using a keyboard in Braille mode in RUDO system, which can also be combined with a refreshable Braille display, allows a blind person to achieve very good results in writing and subsequent editation. Voice feedback can be used after writing a text. If a typing error is discovered, all that has to be done is to press a button on the refreshable Braille display above the incorrect character and the cursor will automatically move to that character, so the blind person can rewrite it straightaway.

### Appendix A4. Using Braille on a Keyboard in Braille Mode in RUDO

For illustration, given below are the six-dot and eight-dot Braille characters and the way they are typed by using selected keys on the keyboard, as used in Braille mode in RUDO.

I    II
            ---- ---- 
            |fj| |14| 
            |dk| |25| 
            |sl| |36| 
            ---- ---- 
		

Character I shows the keyboard keys ascribed to Braille dots. In character II Braille dots are marked by an index according to the established convention. I and II represent a six-dot Braille character.

III  IV
            ---- ---- 
            |fj| |14| 
            |dk| |25| 
            |sl| |36| 
            |ma| |78| 
            ---- ---- 
		

Character III shows the keyboard keys ascribed to Braille dots, “m” represents the spacebar. In character IV Brailled dots are marked by an index according to the established convention. III and IV represent an eight-dot Braille character. Braille is slightly different in each language and the version given below is that which is used in RUDO (i.e., Slovak):
